# The TRPV1-PKM2-SREBP1 axis maintains microglial lipid homeostasis in Alzheimer’s disease

**DOI:** 10.1038/s41419-024-07328-8

**Published:** 2025-01-14

**Authors:** Xudong Sha, Jiayuan Lin, Kexin Wu, Jia Lu, Zhihua Yu

**Affiliations:** Department of Pharmacology and Chemical Biology, Shanghai Jiao Tong University School of Medicine, Shanghai, 200025 China

**Keywords:** Microglia, Alzheimer's disease

## Abstract

Microglia are progressively activated by inflammation and exhibit phagocytic dysfunction in the pathogenesis of neurodegenerative diseases. Lipid-droplet-accumulating microglia were identified in the aging mouse and human brain; however, little is known about the formation and role of lipid droplets in microglial neuroinflammation of Alzheimer’s disease (AD). Here, we report a striking buildup of lipid droplets accumulation in microglia in the 3xTg mouse brain. Moreover, we observed significant upregulation of PKM2 and sterol regulatory element binding protein 1 (SREBP1) levels, which were predominantly localized in microglia of 3xTg mice. PKM2 dimerization was necessary for SREBP1 activation and lipogenesis of lipid droplet-accumulating microglia. RNA sequencing analysis of microglia isolated from 3xTg mice exhibited transcriptomic changes in lipid metabolism, innate inflammation, and phagocytosis dysfunction; these changes were improved with capsaicin-mediated pharmacological activation of TRPV1 via inhibition of PKM2 dimerization and reduction of SREBP1 activation. Lipid droplet-accumulating microglia exhibited increased mitochondrial injury accompanied by impaired mitophagy, which was abrogated upon of TRPV1 activation. Capsaicin also rescued neuronal loss, tau pathology, and memory impairment in 3xTg mice. Our study suggests that TRPV1-PKM2-SREBP1 axis regulation of microglia lipid metabolism could be a therapeutic approach to alleviate the consequences of AD.

## Introduction

Microglia are the brain resident immune cells and maintain brain homeostasis. In the aging brain, microglia lose the homeostatic phenotype and show profound dysfunction including reactive oxygen species (ROS) generation, proinflammatory cytokine production, and dysfunctional phagocytosis [1]. Single-cell transcriptomic studies showed several subpopulations of microglia in aging and disease, such as the protective phagocytic population “disease-associated microglia” [2], and the dysfunctional phenotype “neurodegenerative microglia” [3].

Lipid droplets are accepted as inflammatory markers in response to stress and inflammation in myeloid cells, including eosinophils in allergic inflammation, macrophages in atherosclerotic lesions, and leukocytes in inflammatory arthritis. As lipid-storing organelles, lipid droplets are the sites of eicosanoids and inflammatory cytokine production and storage and further participate in antigen presentation. Lipid droplets have been detected in a lipopolysaccharide-stimulated hippocampal slice of mouse and in the microglia cell line [4, 5]. A novel state of lipid droplet-accumulating microglia was identified in the aging mouse and human brain. These lipid droplet-accumulating microglia in the aging brain exhibit a characteristic transcriptional signature including phagocytosis defects, and increased ROS and proinflammatory cytokines [6]. In human induced pluripotent stem cell-derived microglia, Aβ induces acyl-CoA synthetase long-chain family member 1 expression, triglyceride synthesis and lipid droplet accumulation in Alzheimer’s disease (AD) having the *APOE4/4* genotype [7]. However, little is known about the formation and role of lipid droplets in microglial neuroinflammation of AD.

Sterol regulatory element binding proteins (SREBP) regulate essentially all genes involved lipogenesis and lipid uptake including cholesterol, triglycerides, phospholipids, and fatty acids [8]. There are three SREBP proteins: SREBP1a, SREBP1c, and SREBP2. SREBP1a is a potent activator of all SREBP responsive genes. SREBP1c and SREBP2 are activators of fatty acid and cholesterol synthesis, respectively [9]. Newly synthesized SREBPs are associated with SREBP-cleavage activating protein (SCAP) and are located on the endoplasmic reticulum (ER) membrane. SCAP-SREBP interacts with insulin-induced genes on the ER when cellular sterol levels are sufficient. SCAP-SREBP is escorted from the ER to the Golgi; active SREBPs are then released and translocated to the nucleus to regulate gene expression when sterol levels drop.

Pyruvate kinase (PK) catalyzes phosphoenolpyruvate (PEP) to pyruvate, the last step of glycolysis. PK exists in four different isoforms: PKM1, PKM2, PKL, and PKR. PKM2 is highly expressed in the lung, brain, and intestines and is the predominant form of proliferative state cells [10]. As the rate-limiting glycolytic enzyme, PKM2 catalyzes the phosphoryl group of PEP to ADP, thus generating ATP. Stimulating PKM2 to PKM1, or inhibiting formation of the PKM2 dimer, results in a metabolism switch from aerobic glycolysis to oxidative phosphorylation [11, 12]. PKM2 was shown to activate SREBP target genes by interacting with nuclear SREBP1a (nSREBP1) in HepG2 cells [13]. The PKM2-TMEM33 axis regulates SREBP1 activation and lipid homeostasis in cancer cells by controlling SCAP stability [14]. Capsaicin ameliorates inflammation by inhibiting the PKM2-L-lactate dehydrogenase A-mediated Warburg effect in sepsis [15]. Oxidized low-density lipoprotein stimulation induced interaction of PKM2-SREBP1 and fatty acid synthase (FASN) expression [16].

Here, we report lipid droplet accumulation and neuroinflammation in microglia in the 3xTg mouse brain. PKM2 dimerization was necessary for SREBP1 activation and lipogenesis of lipid droplet-accumulating microglia. RNA sequencing analysis of microglia isolated from 3xTg mice exhibited transcriptomic changes in lipid metabolism, innate inflammation, and defective phagocytosis; these effects were improved with capsaicin-mediated pharmacological activation of TRPV1 via inhibition of PKM2 dimerization and reduction of SREBP1 activation. Capsaicin also rescued neuronal loss, tau pathology, and memory impairment in 3xTg mice. Our study suggests that TRPV1-PKM2-SREBP1 axis regulation of neuronal and microglia lipid metabolism could be a therapeutic approach to alleviate the consequences of AD.

## Methods

### Human brain samples

Human postmortem tissues from both control subjects and AD patients were obtained from the Netherlands Brain Bank, which is part of the Netherlands Institute for Neuroscience in Amsterdam, Netherlands. The Netherlands Brain Bank had secured written informed consent for the use of brain autopsy specimens for research purposes following the subjects’ deaths. Immunofluorescence assays were performed on 8 micron sections using antibodies specific to Iba-1, GFAP, PKM2, and SREBP1, as detailed in the following procedures.

### Mice

3xTg transgenic mice (no. 003378) were purchased from The Jackson Laboratory (Bar Harbor, ME, United States). Roughly equal numbers of male and female mice were used for the in vivo experiments. Age- and sex-matched wild-type (WT) C57BL/6 mice were obtained from Shanghai SLAC Laboratory Animal Co., Ltd. (Shanghai, China). The mice were housed at room temperature (RT, 22 ± 1 °C) under a 12 h light/dark cycle. The protocols of all animal experiments were approved by the Animal Experimentation Ethics Committee of Shanghai Jiao Tong University School of Medicine (Shanghai, China).

### Capsaicin Treatment

Capsaicin was purchased from Target Molecule (T1062, TargetMol, Shanghai, China). Seven-month-old male and female littermate WT and 3xTg mice were injected with capsaicin (1 mg/kg, intraperitoneally; a single injection/day for 1 month) [17, 18]. Behavioral tests were performed 24 h after the last capsaicin injection. Afterward, the mice were euthanized and brain tissues were collected for Western blots and histological analyses.

### Morris water maze

Spatial learning and memory of mice tested by the Morris water maze (MWM) was preformed according to a previous protocol with some modification [19]. Firstly, 1 day before the experiment, the mice were allowed to adapt to the test equipment (120 cm in diameter). The maze was artificially divided into four quadrants and the platform (10 cm in diameter), fixed 1 cm below the water surface, was placed in the center of the third quadrant. Objects of different color and shape were attached to the wall of each quadrant, and used as landmarks. During the training period, the mice were allowed to swim freely for 60 s to find the platform. Mice that did not find the platform were guided to it and allowed to stay in the platform for 10 s. The mice were trained four times per day for 5 days and data are the average of the four trials. After the last training trial, the platform was removed and the memory retention of mice was tested in the probe trail. Using the overhead camera to monitor the swimming activities of each mouse, and automatically recorded through the maze behavior tracking software (EthoVision XT16).

### Traction test

The traction test was used to measure muscle strength and equilibrium [20]. Mice are suspended by their front paws from a horizontal rope ~40–50 cm above the table to provide sufficient time and space for the animal to land. Duration time was recorded and scored according to the following criteria: 0– s, zero points; 5–9 s, one point; 10–14 s, two points; 15–19 s, three points; 20–24 s, four points; 25–29 s, five points; and >30 s, six points.

### Y-Maze test of spontaneous alternation

The spontaneous alternation trail was performed in a symmetrical black Y-Maze with three arms (30 cm × 10 cm × 10 cm) at 120 angles, designated A, B, and C. The mice were placed in the center of the Y-Maze and allowed to probe the maze for 5 min. A video camera mounted above the maze recorded the movements of the mice for analysis.

### Immunofluorescence

Human brain sections embedded in paraffin were deparaffinized with xylene, followed by antigen retrieval with sodium citrate. Coronal sections of mice brain were sliced using a cryostat (Leica) and stored at −20 °C until use. Upon use, the sections were washed with phosphate buffered saline (PBS) and incubated with blocking buffer (0.3% Triton-X-100, 10% normal goat serum in PBS) for 1.5 h at room temperature, followed by incubation with primary antibodies overnight at 4 °C. Primary antibodies used were: IBA (ab178846, 1:200, Abcam, Cambridge, UK), IBA (ab283346, 1:100, Abcam, Cambridge, UK), IBA (GB12105 1:500, Servicebio, Hubei, China), CD68 (ab53444, 1:50, Abcam, Cambridge, UK), Plin2 (ab108323, 1:500, Abcam, Cambridge, UK), PKM2 (D78A4, 1:50, Cell Signaling Technology, Massachusetts, USA), PKM2 (GB11392 1:50, Servicebio, Hubei, China), PKM2 (sc-365684 1:100, Santa Cruz, California, USA), GFAP (BD12096 1:500, Servicebio, Hubei, China), AT8 (GB113883 1:500, Servicebio, Hubei, China), NeuN (ab279296, 1:1000, Abcam, Cambridge, UK), NeuN (AF1072 1:100, Beyotime, Shanghai, China), 4G8 (800701 1:500, BioLegend, SanDiego, USA), SREBP1 (AF8055 1:50, Beyotime, Shanghai, China), SREBP1 (sc-365513 1:100, Santa Cruz, California, USA), PARKIN (AF7680, 1:50, Beyotime, Shanghai, China). After primary antibody incubation, sections were washed twice with PBS and incubated in PBS containing 5% normal goat serum, 0.3% Triton X-100 and the following secondary antibodies: goat anti-mouse Alexa Fluor 488 (A32723 1:1000, Invitrogen, MA, USA), goat anti-rabbit Alexa Fluor 555 (A32732 1:1000, Invitrogen, MA, USA), goat anti-rabbit Alexa Fluor 647 (A32733 1:1000, Invitrogen, MA, USA), goat anti-mouse Alexa Fluor 647 (A32728 1:1000, Invitrogen, MA, USA), and goat anti-rat Alexa Fluor 647 (Abcam 1:1000, 150159, Cambridge, UK) for 2 h at room temperature. For BODIPY staining, sections were incubated in PBS with BODIPY 493/503 (1 μg/mL, D3922 1:1000, Invitrogen, MA, USA) for 15 min at room temperature. Immunofluorescence images were visualized and captured using a Leica TCS SP8 confocal laser scanning system (Leica Microsystems, Wetzlar, Germany).

### Microglial morphological analysis

Two-dimensional morphological analysis of microglia was performed using ImageJ software as described previously [21]. Firstly, 50 microglial cells per group randomly selected from the cerebral cortex were segmented into single cells using a custom-written ImageJ plugin. Single-cell images were automatically converted to 8 bit and transformed into binary images by application of an automatically calculated intensity threshold. The parameters “Number of branches”, “Ramification Index”, and “Total tree length” were quantified using the FIJI plugins “Skeletonize” and “Analyze skeleton 2D/3D” to skeletonize and analyze the binary single-cell images obtained in the previous step.

### RNA-seq analysis

Affinity purification of translating RNA, amplification, and sequencing library construction were described as previously [22]. Samples are sequenced on the platform to get image files and the original data in FASTQ format is generated. The reference genome and gene annotation files were downloaded from genome website in data mapping analysis. Reference genome index was built by Bowtie2 (2.2.6) and the filtered reads were mapping to the reference genome using TopHat2 (2.0.14), the default mismatch was no >2. The alignment region distribution of mapped reads was calculated. For mRNA analysis, we used HTSEq. (0.9.1) statistics to compare the Read Count values on each gene as the original expression of the gene, and then used Fragments Per Kilobase of exon model per Million mapped fragments to standardize the expression. The DESEq. (1.30.0) was used to analyze the genes of difference expression with screened conditions as follows: expression difference multiple |log_2_ fold change | > 1, significant *p*-value < 0.05. R language Pheatmap (1.0.8) software package was used to perform bi-directional clustering analysis of all different genes of samples. We get heat map according to the expression level of the same gene in different samples and the expression patterns of different genes in the same sample with Euclidean method to calculate the distance and Complete Linkage method to cluster. Next, the total detected genes in 3xTg versus WT or 3xTg+CAP versus 3xTg were analyzed by gene set enrichment analysis (GSEA) (version 4.03) using hallmark gene sets (version 7.1) and of Kyoto Encyclopedia of Genes and Genomes (KEGG) subset of canonical pathways (version 7.0) in GSEA databases with total 236 gene sets.

### Preparation of Aβ_1-42_ oligomer

Aβ_1–42_ (A-42-T-1, Genicbio, Shanghai, China) was dissolved in 1,1,1,3,3,3-hexafluoro 2-propanol (Sigma-Aldrich Corporation, St. Louis, MO, USA) at 1 mg/ml and stored at −20 °C as described previously [23]. For preparation of the Aβ_1–42_ oligomer, the dried monomeric peptide Aβ was dissolved in ddH_2_O at a concentration of 100 mM and incubated at 4 °C for 24 h.

### BV2 cell culture

BV2 cells were cultured in Dulbecco’s modified Eagle’s medium (DMEM, L110KJ, BasalMedia, Shanghai, China) containing 10% fetal bovine serum (S711-001S, Lonsera, Uruguay), 2 mM L-glutamine (C0212, Beyotime, Shanghai, China), 100 U/mL penicillin and 0.1 mg/ml streptomycin (C0222, Beyotime, Shanghai, China) under standard culture conditions (37 °C with 5% CO_2_ and 95% relative humidity). BV2 cells were seeded at 3 × 10^5^ cells/well on poly-L-lysine (PLL) coated 6-well plates. After 12 h, the cells were preincubated with 10 μM capsaicin, 0.5 μM NADA (T62530, TargetMol, Shanghai, China), 1 μM MSP-3 (T28118, TargetMol, Shanghai, China), 1 μM shikonin (T1125, TargetMol, Shanghai, China) or 1 μM GW3965 (T6310L, TargetMol) for 0.5 h and then 2 μM Aβ_1-42_ was added for 24 h.

### Transfection of BV2 cells with siRNA

BV2 cells were seeded at 2 × 10^5^ cells/well on PLL-coated 6-well plates and transfected with siRNAs targeting PKM2 (sequences are listed in Table S1) using Lipo8000™ Transfection Reagent (C0533-1.5 ml, Beyotime, Shanghai, China) according to the manufacturer’s instructions. Scrambled siRNA and target gene-specific siRNAs were purchased from Obio Technology (Shanghai, China).

### Quantification of cellular uptake of Aβ_1-42_

BV2 cells were plated in a PLL-coated 96-well plate at a density of 1 × 10^4^ cells/well with DMEM containing 10% fetal bovine serum and cultured for 24 h to allow for cell attachment. Following treatment, the cells were incubated with 2 μg/ml fluorescein isothiocyanate (FITC)-conjugated Aβ_1–42_ (Aβ_1–42_-FITC) in serum-free DMEM. Afterward, the cells were washed with 0.01% PBS, fixed with 4% paraformaldehyde, and stained with DAPI. Cellular uptake of Aβ_1–42_-FITC was quantitatively analyzed using a Cellomics KineticScan reader (Thermo Fisher Scientific), as described previously [24].

### BODIPY in vitro staining

BV2 cells were seeded at 7 × 10^4^ cells/chamber on PLL-coated four-chamber glass-bottom dishes. Following treatment, cells were fixed in 4% paraformaldehyde for 20 min, washed three times in PBS, and incubated in PBS with BODIPY 493/503 and DAPI (C1002 1:1,000, Beyotime, Shanghai, China) for 10 min at room temperature. Dishes were washed three times in PBS, and the last time was retained. Five randomly selected visual fields per chamber were photographed (63 × magnification) using a Leica TCS SP8 confocal laser scanning system.

### Quantitative reverse-transcription polymerase chain reaction (qRT-PCR)

Cell samples were homogenized in TRIzol reagent (R0016, Beyotime, Shanghai, China), RNA was extracted, reverse transcribed to complementary DNA with PrimeScript™ II 1st Strand cDNA Synthesis Kit (6210 A, TAKARA, Shiga, Japan), and subjected to qPCR analysis (Light Cycler 480 II; Roche) as previously described^(24)^. Primer sequences (Sango Biotech) are listed in Table S1.

### Immunoprecipitation

Total protein lysates were obtained in NP-40 (P0013F, Beyotime, Shanghai, China). Total protein concentration of the supernatants was quantified using an Enhanced BCA Protein Assay Kit (P0010S, Beyotime, Shanghai, China). A total of 1 mg of protein was mixed with 1 μg of the primary antibody or IgG (A7028, Beyotime, Shanghai, China), and the mixture was rotated on a shaker at 4 °C overnight. Beads (sc-2003 Protein A/G PLUS-Agarose, Santa Cruz, TX, USA) were added to the mixture and shaken at 4 °C for 3 h. Then, the beads were collected by centrifugation (1000 rpm) and washed three times with NP-40. 2 × sample buffer (P0015B, Beyotime, Shanghai, China) was added to the beads before boiling for 10 min. The supernatant was collected and subsequent steps were performed as described for western blotting.

### Western blotting

Western blotting was performed with the similar approach as described in previously reported [24]. Brain tissues or cells extracts were obtained with RIPA lysis buffer (P0013B, Beyotime, Shanghai, China) supplemented with protease inhibitor cocktail (ST505, Beyotime, Shanghai, China). Nuclear protein extracts were prepared with the kit (P0027, Beyotime, Shanghai, China). After heat denaturation, protein in the lysates were separated by Sodium dodecyl sulfate polyacrylamide gel electrophoresis and then transferred to a PolyVinylideneFluoride membrane (IPVH00010, Millipore, Massachusetts, USA). The membranes were blocked with 5% non-fat milk in tris-buffered saline containing 0.1% Tween-20 for 1 h at room temperature followed by incubation with the indicated primary antibodies at 4 °C overnight. The membranes were then incubated with secondary antibodies conjugated to horseradish peroxidase. The following primary antibodies were used: SREBP1 (AF8055 1:1000), β-actin (AA128 1:1000, Beyotime, Shanghai, China), H4 (AF7107, Beyotime, Shanghai, China), PKM2 (GB11392, Servicebio). The band intensities were quantified with ImageJ software.

### Immunocytofluorescence

The BV2 cells were seeded at 7 × 10^4^ cells/chamber on PLL-coated four-chamber glass-bottom dishes. Following specific treatments, cells were fixed in 4% paraformaldehyde for 20 min, washed three times in PBS and incubated with blocking buffer (0.3% Triton-X-100, 10% normal goat serum in PBS) for 1.5 h at room temperature, followed by incubation with primary antibodies overnight at 4 °C. Primary antibodies used were: TOMM20 (AF1717, 1:50, Beyotime, Shanghai, China), PARKIN (AF7680, 1:50, Beyotime, Shanghai, China), CD68 (ab53444, 1:50, Abcam, Cambridge, UK), p-PKM2 (11456, 1:50, Signalway Antibody, Shanghai, China), H4K12la (PTM-1411RM, 1:50, PTM-Bio, Hangzhou, China). After primary antibody incubation, BV2 cells were washed twice with PBS and incubated in PBS containing 5% normal goat serum, 0.3% Triton X-100 and the following secondary antibodies: goat anti-mouse Alexa Fluor 488 (A32723, 1:1000, Invitrogen, MA, USA), goat anti-rabbit Alexa Fluor 555 (A32732, 1:1000, Invitrogen, MA, USA) and goat anti-rat Alexa Fluor 647 (Abcam, 1:1000, 150159, Cambridge, UK) for 2 h at room temperature. Immunofluorescence images were visualized and captured using a Leica TCS SP8 confocal laser scanning system.

### Cross-linking reaction

Cell samples were collected, washed twice with PBS and incubated in PBS pH 8.0 + 1 mM disuccinimidyl suberate (C100015, Sango Biotech, Shanghai, China) for 30 min at room temperature as previously described [25], and subsequent steps were performed as described in the western blotting.

### Assessment of mitophagy

The BV2 cells were seeded at 7 × 10^4^ cells/chamber on PLL-coated four-chamber glass-bottom dishes. To assess mitophagy, BV2 cells were transduced with adenovirus carrying COX8-mt-mkeima (MOI ratio = 200:1, Obio technology, Shanghai, China) for 24 h. Following specific treatments, cells were washed three times in PBS, and the last time was retained. Five randomly selected visual fields per chamber were photographed (63 × magnification) using a Leica TCS SP8 confocal laser scanning system. This construct localizes to mitochondria and emits fluorescence differently under neutral or acidic conditions. An increase in the red signal indicated elevated levels of mitophagic flux. Mitophagy levels were quantified by measuring the pixel area in the red channel (acidic) and normalizing it to the signal in the green channel (neutral). Calculations were conducted using data from six randomly selected areas.

### Assessment of autophagy

The BV2 cells were seeded at 5 × 10^4^ cells/chamber on PLL-coated four-chamber glass-bottom dishes. To visualize the extent of autophagosome delivery to lysosomes, we transfected BV2 cells with mRFP‐GFP‐tagged LC3 plasmid (Addgene, Watertown, MA, USA) for 48 h. Following specific treatments, cells were fixed in 4% paraformaldehyde for 20 min, washed three times in PBS and incubated in PBS with DAPI (C1002 1:1,000, Beyotime, Shanghai, China) for 10 min at room temperature. Dishes were washed three times in PBS, and the last time was retained. Five randomly selected visual fields per chamber were photographed (63 × magnification) using a Leica TCS SP8 confocal laser scanning system. Within lysosomes, the fluorescent signal from GFP was quenched, while mRFP showed no significant change under acidic conditions. Autophagosomes were observed as yellow dots (mRFP^+^ GFP^+^), whereas autolysosomes were observed as red dots (mRFP^+^ GFP^-^).

### Measurement of mitochondrial DNA

The BV2 cells were seeded at 5 × 10^4^ cells/chamber on PLL-coated four-chamber glass-bottom dishes. Following specific treatments, BV2 cells were first treated with MitoTracker Red (40741ES50, 200 nM, Yeasen Biotechnology, Shanghai, China) for 40 min, followed by incubation in Picogreen (12641ES01, 500 ng/ml, Yeasen Biotechnology, Shanghai, China) for 15 min. Subsequently, cells were imaged using a Nikon microscope equipped with a 63 × oil-immersion objective. To eliminate nuclear DNA signal, the Picogreen^+^ nuclear area was subtracted using ImageJ software. According to a previous protocol [26], the relative intensity of cytosolic mtDNA was represented by the ratio of intensity of green/red channel post nuclei area deduction. Signal intensity in the regions of interest was quantitatively analyzed using ImageJ, based on six randomly selected areas.

### Measurement of mitochondrial membrane potential

Mitochondrial membrane potential was measured by JC‐1 (C2005, Beyotime, Shanghai, China). BV2 Cells were incubated with 10 μM JC‐1 at 37 °C for 20 min. The fluorescence level was assessed by TCS SP8 confocal laser scanning microscope. The excitation wavelength was 488 nm for monomer and 561 nm for aggregates, while emission wavelength was 530-600 nm. Results were calculated as red/green ratio at ImageJ as reported [27].

### Measurement of mitochondrial ROS

The BV2 cells were seeded at 7 × 10^4^ cells/chamber on PLL-coated four-chamber glass-bottom dishes. Following specific treatments, BV2 cells were treated with Mitochondrial Superoxide Assay Kit (S0061S, 1 μM, Beyotime, Shanghai, China) to measure generation of mitochondrial ROS.

### Lipid peroxidation test

Microglia were seeded and cultured overnight in glass-bottom dishes at a density of 1 × 10^5^ cells/well. Lipid peroxidation was measured using a Lipid Peroxidation Kit (S0043S; Beyotime Institute of Biotechnology). According to the manufacturer’s instructions, microglia were incubated with a 2 μM sensor in PBS for 20 min at 37 °C (shielded from light), then washed with PBS before assessment using a Nikon AXR confocal laser scanning microscope. Images were acquired alternately at 581 nm and 488 nm. Lipid peroxidation was quantified as the red/green ratio using ImageJ software.

### Statistical analysis

All data are expressed as mean ± standard error of the mean (SEM). Statistical analyses were performed using GraphPad Prism software (version 8.0.1) and one-way analysis of variance (ANOVA) followed by Tukey’s multiple comparisons test. The statistical parameters can be found in the figures and figure legends. The statistical significance threshold was set at *p* < 0.05. Detailed information on the sample size, number of replicates, and statistical tests used for each experiment is provided in the figure legends.

## Results

### Correlation of lipid droplet accumulation and expression of PKM2/SREBP1 axis in AD microglia

**Fig. 1:**
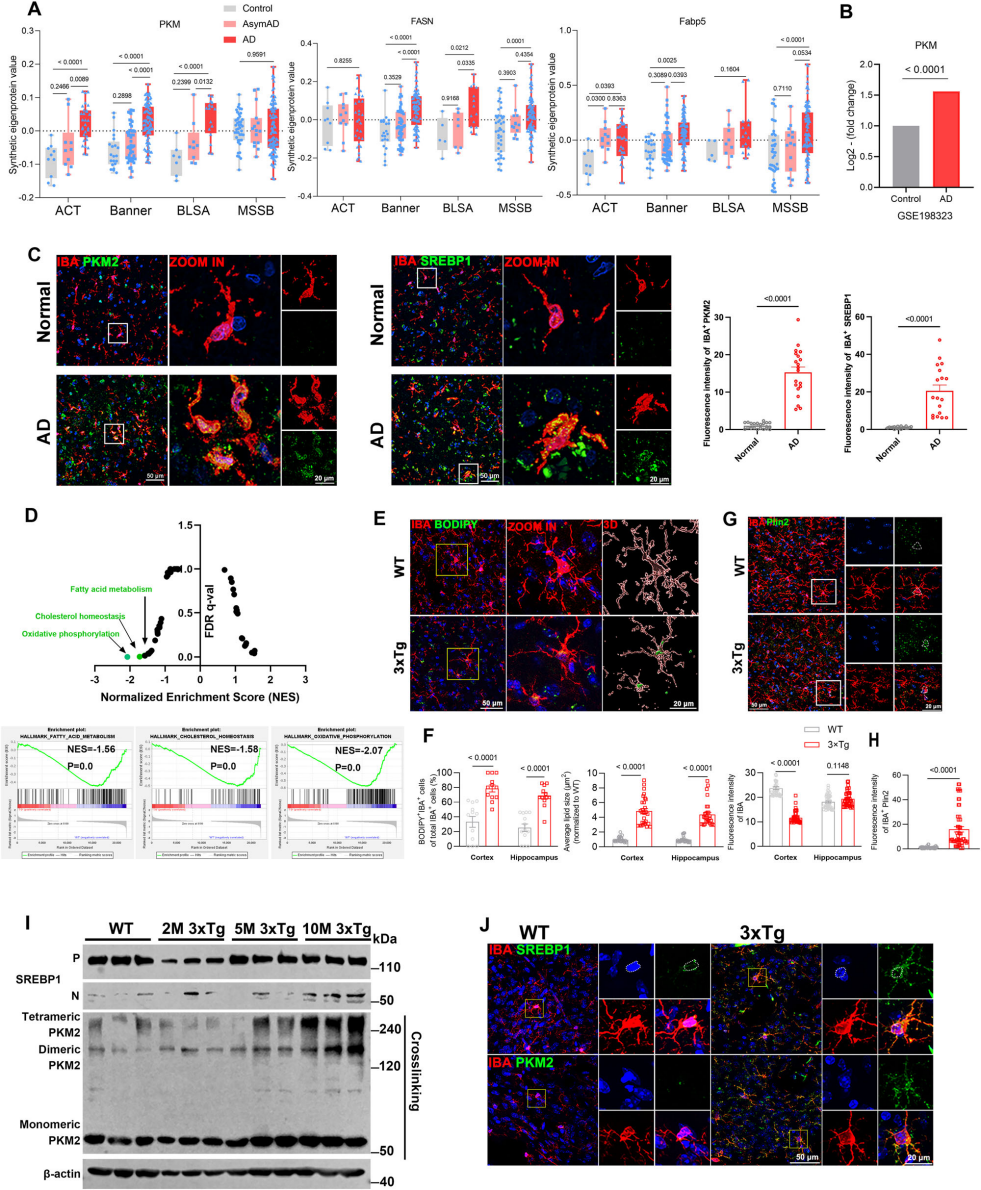
Correlation of lipid droplet accumulation and expression of PKM2/SREBP1 axis in AD microglia. **A** Analysis of protein levels in brain tissue from public Large-scale proteomic data. Control, AsymAD, and patients with AD (*n* = 453) were measured by label-free MS and analyzed by weighted correlation network analysis and differential abundance. Brain tissue was analyzed from postmortem dorsolateral prefrontal cortex in the BLSA (*n* = 11 Control, n = 13 AsymAD, *n* = 20 AD, *n* = 44 total), Banner (*n* = 26 Control, n = 58 AsymAD, *n* = 94 AD, *n* = 178 total), MSSB (*n* = 46 Control, *n* = 17 AsymAD, *n* = 103 AD, *n* = 166 total) and ACT (*n* = 11 Control, *n* = 14 AsymAD, *n* = 40 AD, *n* = 65 total). **B** Analysis of *pkm* expression in Control and AD from public snRNA-seq data (GSE198232). **C** Representative immunofluorescent images of SREBP1 or PKM2 co-stained with IBA in the cerebral cortex of normal and AD individuals, with quantification of pkm2 and SREBP1 intensity in microglia (*n* > 20 cells per group). **D** Gene set enrichment analysis of the top enriched gene signatures associated with 3xTg versus WT mice. Hallmark 50 gene sets were used for this analysis. Oxidative phosphorylation and cholesterol homeostasis, fatty acid metabolism signature was decreased in 3xTg versus WT mice. The above signatures were generated by GSEA (v4.1.0) with adjusted *P*-value and normalized enrichment score. **E**, **F** Representative images of BODIPY co-stained with IBA in the cortex of 8 month-old WT and 3xTg mice (*n* = 6 mice per group). **G**, **H** Representative images of Plin2 co-stained with IBA in the cortex of mice, with quantification of plin2 intensity in microglia (*n* = 5 mice per group). **I** Western blotting analysis of nSREBP1/pSREBP1, monomeric, dimeric, and tetrameric PKM2 in the cortex of mice (*n* = 3 mice per group). **J** Representative images of SREBP1 or PKM2 co-stained with IBA in the cortex of mice. AsymAD asymptomatic AD, ACT Adult Changes in Thought Study, Banner Banner Sun Health Research Institute, BLSA Baltimore Longitudinal Study of Aging, MSSB Mount Sinai School of Medicine Brain Bank, FASN fatty acid synthase, FABP5 fatty acid binding protein 5.

To rapidly acquire energy to perform immune functions, microglia tend to undergo metabolic reprogramming from oxidative phosphorylation to aerobic glycolysis in neurodegenerative diseases [27]. PKM2 is a key rate-limiting enzyme for aerobic glycolysis, and recent studies have revealed that microglia surrounding amyloid plaques in 5xFAD mice express high levels PKM2 [28]. Accordingly, we analyzed public large-scale proteomic data [29] and RNA-seq data (GSE198323) and found that PKM was markedly elevated in the brain of AD (Fig. 1A, B). The public large-scale proteomic data contained dorsolateral prefrontal cortex tissues of 65 cases from the Adult Changes in Thought Study (ACT), 178 cases from the Banner Sun Health Research Institute (Banner), 44 cases from the Baltimore Longitudinal Study of Aging (BLSA), and 166 cases from the Mount Sinai School of Medicine Brain Bank (MSSB), for a pool of 453 control, asymptomatic AD (AsymAD), and AD brains (Extended Data Fig. 1A, B). Additionally, FASN, a target gene of SREBP1a, and fatty acid binding protein 5, essential enzymes in fatty acid synthesis and transport pathways, were significantly increased in AD brains (Fig. 1A). Additionally, we performed co-staining on postmortem brain cortex tissue sections from individuals with AD and their age-matched controls, using antibodies against PKM2 and SREBP1, along with the microglial marker Iba-1. Confocal microscopy images revealed significant expression of PKM2 and SREBP1 in microglia within the brains of AD patients (Fig. 1C).

To investigate the role of lipid metabolism in AD, we first analyzed our publicly available bulk RNA-sequencing (RNA-seq) data and performed gene set enrichment analysis (GSEA) of the triple-transgenic model (3xTg) of AD harboring the human APPswe, tauP301L, and PS1M146V mutations (accession no. GSE54679) [30, 31]. The bulk RNA-seq data of the cortex and hippocampus of 8 month-old WT and 3xTg mice was analyzed by GSEA with hallmark 50 gene sets. Our data indicated that oxidative phosphorylation, cholesterol homeostasis, and fatty acid metabolism signature was among the top downregulated pathways in 3xTg mice compared to WT mice (Fig. 1D).

We observed characteristic lipid droplets in the cerebral cortex and hippocampus of 3xTg mice but rarely in WT mice (Fig. 1E). Histological staining of Iba-1^+^ microglia with BODIPY, a dye that specifically labels neutral lipids and is commonly used to detect lipid droplets [32], showed that lipid droplets were significantly accumulated in microglia (Fig. 1E, F) in 3xTg mice compared with WT mice. Furthermore, we confirmed the presence of lipid droplet accumulation in microglia of 3xTg mice using immunofluorescence co-staining with the lipid droplet-coated protein plin2 and Iba-1 (Fig. 1G, H).

When cellular sterol levels are adequate, SCAP prompts the sequestration of the inactive precursor form of SREBP1 (pSREBP1) on the ER membrane by binding to insulin-induced genes. In contrast, when sterol levels decrease, SCAP facilitates the transportation of pSREBP1 from the ER to the Golgi apparatus for proteolytic cleavage. This process releases the active nSREBP1, allowing it to translocate into the nucleus and initiate the transcription of genes associated with synthesis [9]. Moreover, PKM2 has been implicated in the regulation of SREBP1 activation, thereby influencing the lipid de novo synthesis pathway in triple-negative breast cancer [33]. Recent literature reports an increase in PKM2 protein expression in the brains of AD patients as they transition from the latency stage to cognitive dysfunction stage, suggesting that elevated PKM2 protein levels may significantly contribute to cognitive dysfunction in AD patients [34]. The tetrameric form of PKM2 protein exhibits robust metabolic activity, facilitating the conversion of phosphoenolpyruvate to pyruvate within the glycolytic pathway. Conversely, the dimeric form, which is less enzymatically active, possesses the ability to translocate into the nucleus, where it functions as a transcriptional coactivator [35]. We examined the expression of nSREBP1/pSREBP1, monomeric, dimeric, and tetrameric PKM2 in the cortex of WT, as well as 2-month-old, 5-month-old, and 10-month-old 3xTg mice, using western blotting. The experimental results revealed a continuous upregulation in the expression of nSREBP1/pSREBP1, monomeric, dimeric, and tetrameric PKM2 with increasing age in 3xTg mice (Fig. 1I, Extended Data Fig. 1C). Furthermore, we performed immunofluorescence co-staining of PKM2 or SREBP1 along with markers for microglia. The signal intensity of PKM2 and SREBP1 was significantly elevated in the microglia of 3xTg mice compared to WT mice (Fig. 1J, Extended Data Fig. 1D). However, we did not observe any significant differences in PKM2 or SREBP1 intensity in astrocytes between 3xTg and WT mice (Extended Data Fig. 1E). These in vivo findings support the correlation between the activation of SREBP1 and the increased expression of PKM2 in the brains of 3xTg mice.

### Inflammation-activated microglia of 3xTg mice exhibited transcriptomic changes in lipid metabolism, immunity, and synapse function

**Fig. 2:**
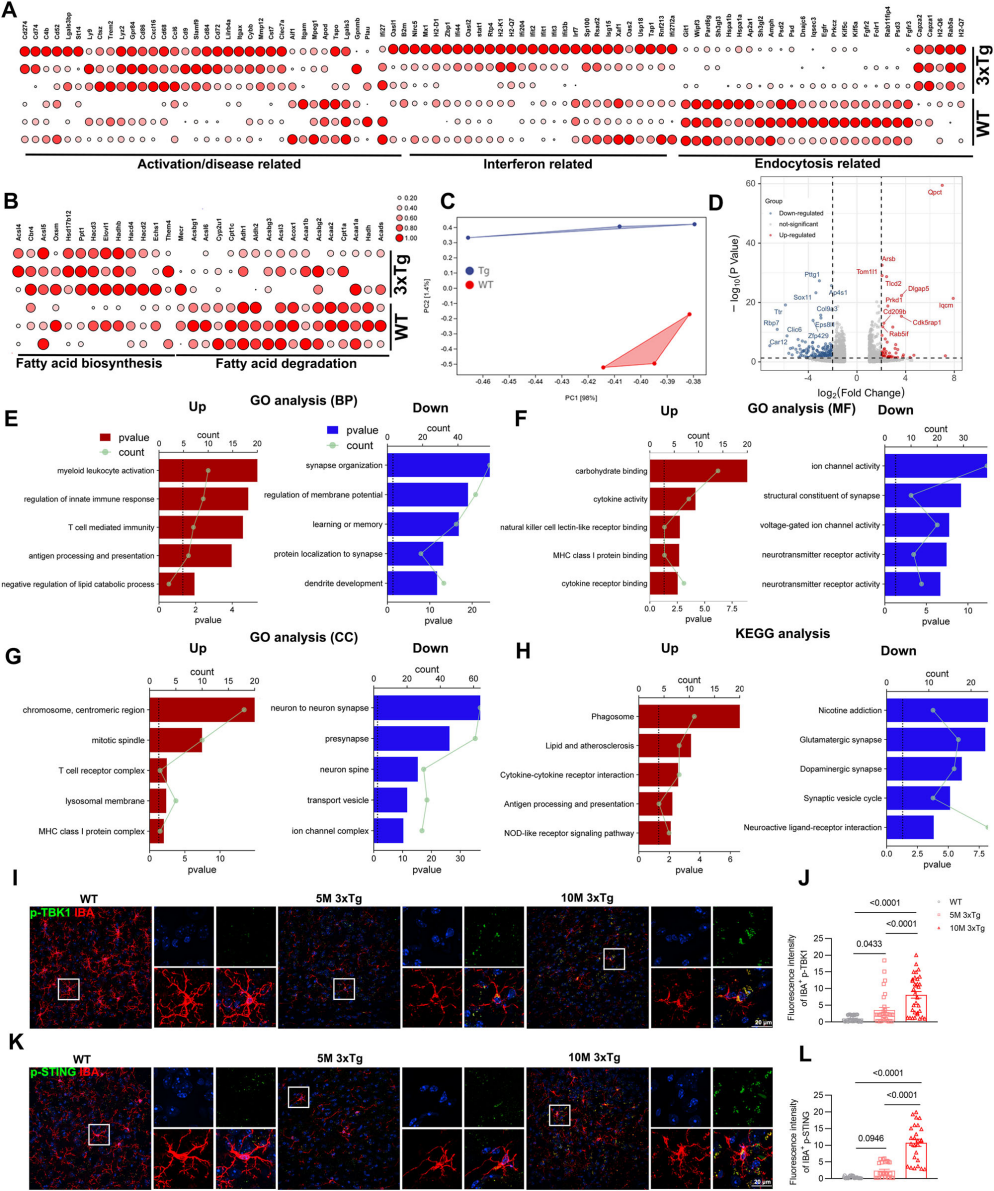
Inflammation-activated microglia of 3xTg mice exhibited transcriptomic changes in lipid metabolism, immunity and synapse function. **A**, **B** Differential gene expression from bulk RNA-seq analysis of microglia isolated from 8 month-old WT and 3xTg mice (*n* = 3 mice per group). The heatmap shows all significant DEGs associated with interferon or activation/disease-related microglia (**A**) and lipid metabolism (**B**). **C**, **D** Principal component analysis of 8-month-old WT and 3xTg mice using the whole transcriptome (“all genes”). Volcano plot of the 895 downregulated DEGs (blue) and 294 upregulated DEGs (red) of 8 month-old WT and 3xTg mice (false discovery rate (FDR) ≤ 0.01, | log_2_[fold change (FC)] | ≥ 2). **E**–**G** The left panel shows the top 5 most enriched GO Biological Process gene sets (**E**) for upregulated DEGs, while the right panel for downregulated DEGs. The same is done for GO Molecular Function gene sets (**F**) and GO Cellular Component gene sets (**G**). **H** The left panel shows the top 5 most enriched KEGG gene sets for upregulated DEGs, while the right panel shows those for downregulated DEGs. **I**–**L** Representative images of p-TBK1 (**I**) or p-STING (**K**) co-stained with IBA in the cortex of WT and 3xTg mice, with quantification of p-TBK1 (**J**) or p-STING (**L**) intensity in microglia (*n* = 5 mice per group). GO Gene Ontology, KEGG Kyoto Encyclopedia of Genes and Genomes. Statistical tests: one-way ANOVA (**J**, **L**) followed by Tukey’s post hoc test. Data represent the mean ± s.e.m.

We performed RNA-seq of microglia isolated from 8-month-old WT and 3xTg mice. Expression of marker genes associated with activation/disease-related or interferon-associated microglia were significantly increased in microglia of 3xTg mice compared to WT mice (Fig. 2A–D). Furthermore, we found that the de novo lipid synthesis pathway was upregulated in microglia of 3xTg mice, whereas the cellular endocytosis and fatty acid degradation pathways were downregulated in microglia of 3xTg mice (Fig. 2A, B). Transcriptional analysis revealed 1189 significantly differentially expressed genes (DEGs), with 895 downregulated and 294 upregulated DEGs (Fig. 2E, F). Gene Ontology (GO) analysis showed that the upregulated DEGs were enriched in three different types of GO gene sets related to the immune system and cellular responses (Fig. 2E–G). The downregulated DEGs were enriched in three different types of GO gene sets relating to synaptic modification, voltage-gated ion channels, and learning/memory (Fig. 2E–G). Kyoto Encyclopedia of Genes and Genomes (KEGG) analysis showed that the upregulated DEGs were enriched in KEGG gene sets related to lipid metabolism, immune system, and inflammation response (Fig. 2H). The downregulated DEGs were enriched in KEGG gene sets related to multiple neural synapses (Fig. 2H). Taken together, the inflammatory hyperactivation of microglia may be closely related to its abnormal lipid metabolism, which in turn mediates the infiltration of peripheral immune cells.

The presence of type I IFN markers in microglia has increasingly been linked to aging and neurodegenerative diseases across various species [26, 36–38]. Recent studies have highlighted a significant elevation in the levels of p-TBK1 and p-STING proteins in interferon-associated microglia [39]. To confirm the significant increase in IFN-associated microglia in the brains of 3xTg mice, histological staining was conducted with p-TBK1 or p-STING along with IBA. The results showed that the intensity of p-TBK1 and p-STING in microglia was continuously upregulated with increasing age of 3xTg mice (Fig. 2I–L).

This was supported by proteomics data, where 22 significantly upregulated and 17 downregulated proteins were identified in Aβ_1-42_-induced BV2 cells compared with the WT counterparts (Extended Data Fig. 2A, B). GO analysis revealed that the upregulated differentially expressed proteins (DEPs) were enriched in GO cell component gene sets related to the succinate dehydrogenase complex and respiratory chain complex II. On the other hand, the downregulated DEPs were enriched in GO cell component gene sets related to synaptic modification and vesicular transport (Extended Data Fig. 2C). Furthermore, following KEGG analysis, it was revealed that the identified upregulated DEPs were predominantly associated with KEGG gene sets related to lipid metabolism and inflammation response. On the other hand, the downregulated DEPs were found to be mostly enriched in KEGG gene sets associated with lysosome and autophagy (Extended Data Fig. 2D). These results collectively suggest a strong association between abnormal lipid metabolism and dysfunction of inflammation-activated microglia.

### TRPV1/PKM2/SREBP1 axis regulates phagocytosis due to lipid droplet accumulation in Aβ_1-42_-stimulated BV2 cells

**Fig. 3:**
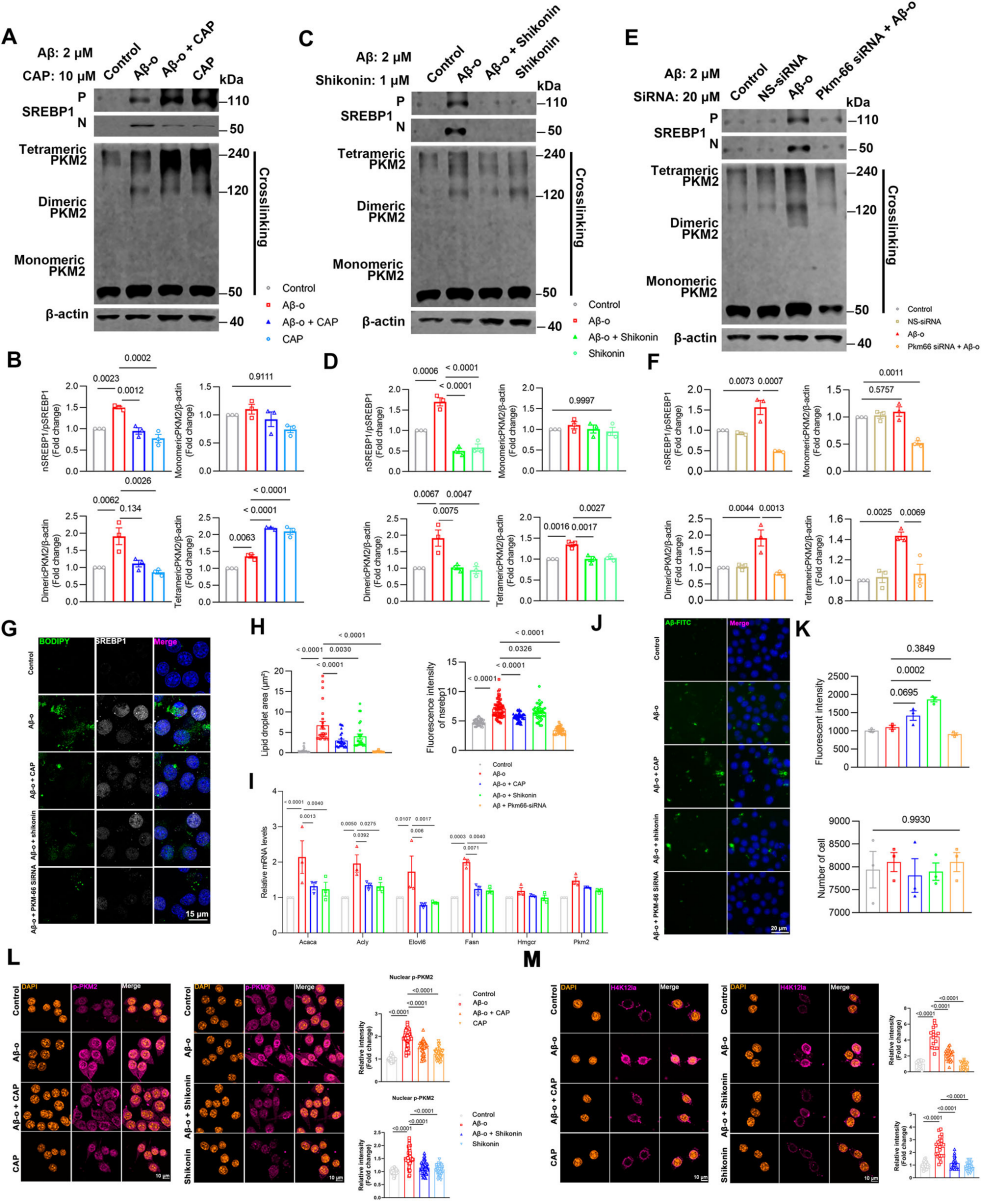
TRPV1/PKM2/SREBP1 axis regulates phagocytosis due to lipid droplet accumulation in Aβ_1-42_-stimulated BV2 cells. **A**–**F** Western blotting analysis and quantification of nSREBP1/pSREBP1, monomeric, dimeric, and tetrameric PKM2 in 2 μM Aβ_1-42-_stimulated BV2 cells for 24 h with 10 μM capsaicin (**A**, **B**), 1 μM shikonin (**C**, **D**), 20 μM Pkm-66 siRNA (**E**, **F**) pretreatment. **G**, **H** Representative images and quantification of nuclear SREBP1 and lipid droplets (*n* = 3, biological replicates). **I** Analysis of *acaca, acly, elovl6, fasn, hmgcr, pkm2* expression (*n* = 3, biological replicates). **J**, **K** Cellular uptake of 2 μg/ml of Aβ_1–42_-FITC by BV2 cells (*n* = 3, biological replicates). **L** Immunostaining and quantification of p-PKM2 in 2 μM Aβ_1-42-_stimulated BV2 cells for 24 h with 10 μM capsaicin or 1 μM shikonin pretreatment (*n* = 3, biological replicates). **M** Immunostaining and quantification of H4K12la in 2 μM Aβ_1-42-_stimulated BV2 cells for 24 h with 10 μM capsaicin or 1 μM shikonin pretreatment (*n* = 3, biological replicates). Statistical tests: one-way ANOVA (**B**–**K**) followed by Tukey’s post hoc test. Data represent the mean ± s.e.m.

As the rate-limiting glycolytic enzyme, PKM2 catalyzes the phosphoryl group of PEP to ADP, thus generating ATP. Stimulating PKM2 to PKM1, or inhibiting PKM2 dimerization, results in a metabolic switch from aerobic glycolysis to oxidative phosphorylation [11, 12, 14]. Capsaicin has been reported to ameliorate inflammation by inhibiting the PKM2-L-lactate dehydrogenase A-mediated Warburg effect in sepsis [15]. We therefore examined the effect of capsaicin on Aβ_1-42_-induced PKM2 and SREBP1 activation. As shown in Fig. 3A, 2 μM Aβ_1-42_ stimulation upregulates dimeric PKM2 and nSREBP1. Our study found that capsaicin significantly increased the level of tetrameric PKM2 in Aβ_1-42_-treated BV2 cells, while the levels of nSREBP1 and dimeric PKM2 decreased (Fig. 3A, B). To further substantiate the influence of TRPV1 channel activity on Aβ_1-42_-induced PKM2 conformational changes and SREBP1 activation, the TRPV1 agonists NADA and MSP-3 were used. The experimental findings demonstrated that both NADA and MSP-3 significantly increased the levels of tetrameric PKM2 in BV2 cells treated with Aβ_1-42_, while concurrently reducing the levels of nSREBP1 and dimeric PKM2 (Extended Data Fig. 3A–D).

We also examined the effect of the PKM2 blocker shikonin on Aβ_1-42_-induced PKM2 conformation and SREBP1 activation. As shown in Fig. 3C, 1 μM shikonin inhibited the expression of both dimeric and tetrameric forms of PKM2 and nSREBP1 in the presence of Aβ_1-42_, while the level of monomeric PKM2 remained unaffected (Fig. 3C, D). Small interfering RNA (siRNA) was used to decrease the expression of PKM2 (Extended Data Fig. 4A, B). BV2 cells were stably transfected with siRNA constructs PKM2-66. Downregulation of PKM2 with PKM2-66 could significantly decrease the activated isoform nSREBP1 and both dimeric and tetrameric forms of PKM2 in the presence of Aβ_1-42_ (Fig. 3E, F). Through immunoprecipitation, we discovered that endogenous PKM2 binds with endogenous nuclear SREBP-1 protein. Additionally, the PKM2-SREBP1 interaction is intensified in Aβ_1-42_-stimulated BV2 cells (Extended Data Fig. 4C).

Capsaicin, shikonin, and siRNA PKM2-66 attenuated the re-localization of SREBP1 from the perinuclear region to the nucleus (Fig. 3G, H). Immunofluorescence showed that capsaicin, shikonin, or siRNA PKM2-66 also decreased BODIPY^+^ puncta in Aβ_1-42_-induced BV2 cells. Aβ_1-42_ increased mRNA levels of SREBP1 target genes (*acaca*, *acly*, *elovl6*, and *fasn*) in microglia compared with control cells; this effect could be inhibited by 10 μM capsaicin or 1 μM shikonin (Fig. 3I). To investigate the implication of the TRPV1-PKM2-SREBP1 axis on microglial phagocytic capacity, BV2 cells were incubated with 2 μg/ml FITC-Aβ_1-42_ for 4 h following pretreatment with 10 μM capsaicin, 1 μM shikonin, or siRNA targeting PKM2 for 24 h (Fig. 3J, K). Phagocytic capacity was increased in the presence of capsaicin, shikonin, and PKM2 knockdown compared with control cells.

The tetramer state of PKM2 has high pyruvic acid kinase activity, while the dimer state has low PK activity. Phosphorylation events, including the phosphorylation of PKM2 at serine 37 (p-PKM2), can reduce the tetramer/dimer ratio, consequently diminish PK activity [40, 41]. p-PKM2 is a key step that initiates its nuclear translocation [42]. Therefore, we investigated the impact of capsaicin and shikonin on Aβ_1-42_-induced p-PKM2 and its nuclear translocation. Figure 3L shows that both capsaicin and shikonin attenuated p-PKM2 and its nuclear translocation induced by stimulation with 2 µM Aβ_1-42_ (Fig. 3L).

Research has shown that lactylation of histone H4 at lysine 12 (H4K12la) enhances PKM2 production, establishing a positive feedback loop among glycolysis, H4K12la, and PKM2, which exacerbates microglial dysfunction [28]. We next investigated the effect of capsaicin and shikonin on Aβ_1-42_-induced H4K12la. As shown in Fig. 3M, 2 µM Aβ1_-42_ significantly upregulated H4K12la, which was reduced by capsaicin and shikonin, suggesting that TRPV1 activation inhibits glycolysis by decreasing H4K12la (Fig. 3M). In conclusion, TRPV1 activation suppresses PKM2 dimer formation by inhibiting PKM2 Ser37 phosphorylation and reduces H4K12la, curbing glycolysis while promoting PKM2 tetramer assembly and oxidative phosphorylation.

### TRPV1/PKM2/SREBP1 axis regulates mitochondrial dysfunction due to lipid droplet accumulation in Aβ_1-42_-stimulated BV2 cells

**Fig. 4:**
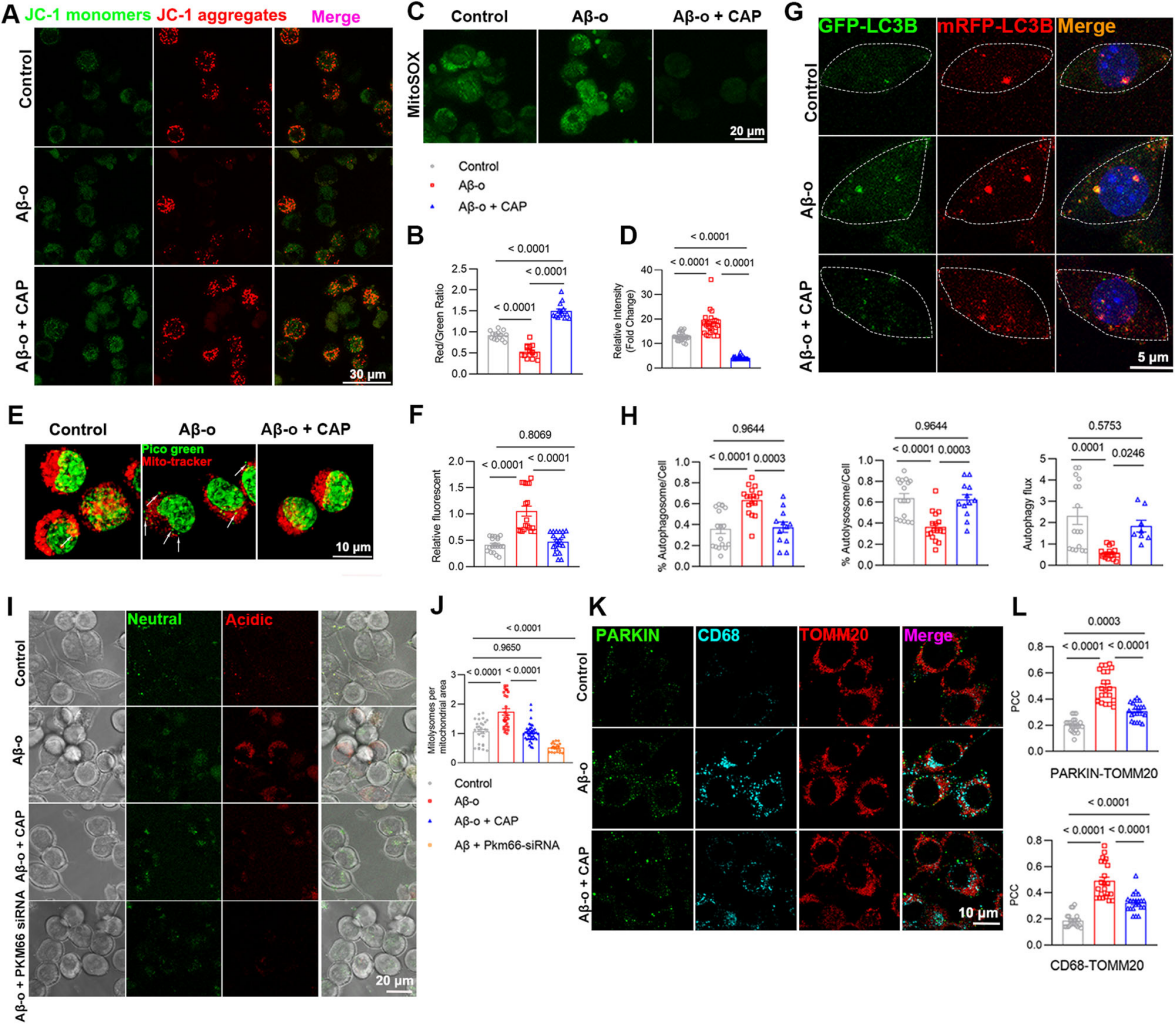
TRPV1/PKM2/SREBP1 axis regulates mitochondrial dysfunction due to lipid droplet accumulation in Aβ_1-42_-stimulated BV2 cells. Representative images of 2 μM Aβ_1-42_-stimulated BV2 cells for 24 h with 10 μM capsaicin pretreatment. **A**–**F** Staining with JC-1 (**A**, **B**), MitoSOX (**C**, **D**), and Mito-tracker Red/Picogreen (**E**, **F**) (*n* = 3, biological replicates). **G**, **H** Representative images and quantification of RFP-GFP-tandem fluorescent LC3-transfected BV2 cells. Quantification of autophagosomes (mRFP- and GFP-positive), autolysosomal (mRFP-positive only) regions, and autophagy flux rates calculated on the basis of RFP/RFP-GFP double positivity only. **I**, **J** Representative images of mt-mKeima-expressing BV2 cells, and analyzed for pixel area in the red channel (Acidic) and normalized to the signal in green channel (Neutral) (*n* = 3, biological replicates). **K**, **L** Immunostained with antibodies for PARKIN (green), CD68 (cyan) and TOMM20 (red). The Pearson’s correlation coefficient (PCC) was calculated from 3 independent experiments (*n* = 3, biological replicates). Statistical tests: one-way ANOVA followed by Tukey’s post hoc test. Data represent the mean ± s.e.m.

In Fig. 3, it is demonstrated that activation of TRPV1 inhibited PKM2 dimer formation and alleviated intracellular phagocytic dysfunction in Aβ_1-42_-stimulated BV2 cells. In addition, previous studies have found that the accumulation of intracellular lipid droplets exacerbates lipid peroxidation and disrupts mitochondrial membrane potential [43, 44]. JC-1 and MitoSOX were used to analyze mitochondria membrane potential or mitochondrial ROS. TRPV1 activation with capsaicin attenuated mitochondria membrane potential depolarization (Fig. 4A, B) and mitochondria ROS production (Fig. 4C, D) in Aβ_1-42_-stimulated BV2 cells. As one of the major components of mitochondria-derived damage-associated molecular patterns, mitochondria release deoxyribonucleic acid (mtDNA) plays an important role in inflammatory responses and cell death [45]. Mito-Tracker and Picogreen probes were used to further validate the role of TRPV1 on mitochondria damage in Aβ_1-42_-stimulated BV2 cells. The results showed that the intracellular mtDNA leakage was significantly downregulated with 10 μM capsaicin pretreatment compared with Aβ_1-42_-stimulated BV2 cells (Fig. 4E, F).

The mRFP-GFP-tagged LC3 reporter was introduced into BV2 cells to monitor the effect of capsaicin on autophagic activity. We observed increased autophagosomes (RFP and GFP) and decreased autolysosomes (RFP only) in Aβ_1-42_-stimulated BV2 cells compared with control group, indicating impaired autophagy flux. Nevertheless, activation of TRPV1 decreased autophagosomes and prompted autolysosomes in Aβ_1-42_-stimulated BV2 cells, indicating restoring autophagy flux (Fig. 4G, H). To better measure mitophagy, we transfected BV2 cells with mitochondrial mKeima, a fluorescent protein that emits signals of different colors at acidic (acidified lysosomes) and neutral (cytoplasmic) pH to measure mitochondrial translocation to lysosomes [26]. We observed an increase in the fluorescent signals from mitolysosomes in Aβ_1-42_-stimulated BV2 cells compared with control group. However, activation of TRPV1 decreased the fluorescent signals from mitolysosomes in Aβ_1-42_-stimulated BV2 cells (Fig. 4I, J). Next, we performed triple-immunostaining with antibodies against PARKIN, CD68 and mitochondrial marker TOMM20. TRPV1 activation decreased the colocalization between PARKIN/TOMM20 or CD68/TOMM20 in Aβ_1-42_-stimulated BV2 cells (Fig. 4K, L). Additionally, both capsaicin and shikonin significantly reduced lipid peroxidation accumulation induced by Aβ_1-42_ stimulation (Extended Data Fig. 5A, B).

### TRPV1 activation rescued memory impairment and reactive microglia in 3xTg mice

Lipid droplet accumulation and neuroinflammation were observed in microglia isolated from microglia-specific TRPV1-gene deficient ApoE4 mice [31]. We monitored whether activation of the TRPV1-PKM2-SREBP1 axis could rescue lipid metabolism in 3xTg mice. Seven-month-old male and female 3xTg mice were treated with 1 mg/kg capsaicin by intraperitoneal injection for 1 month, followed by behavioral assessments, including the Morris water maze (MWM), traction test, and Y-maze test.

**Fig. 5:**
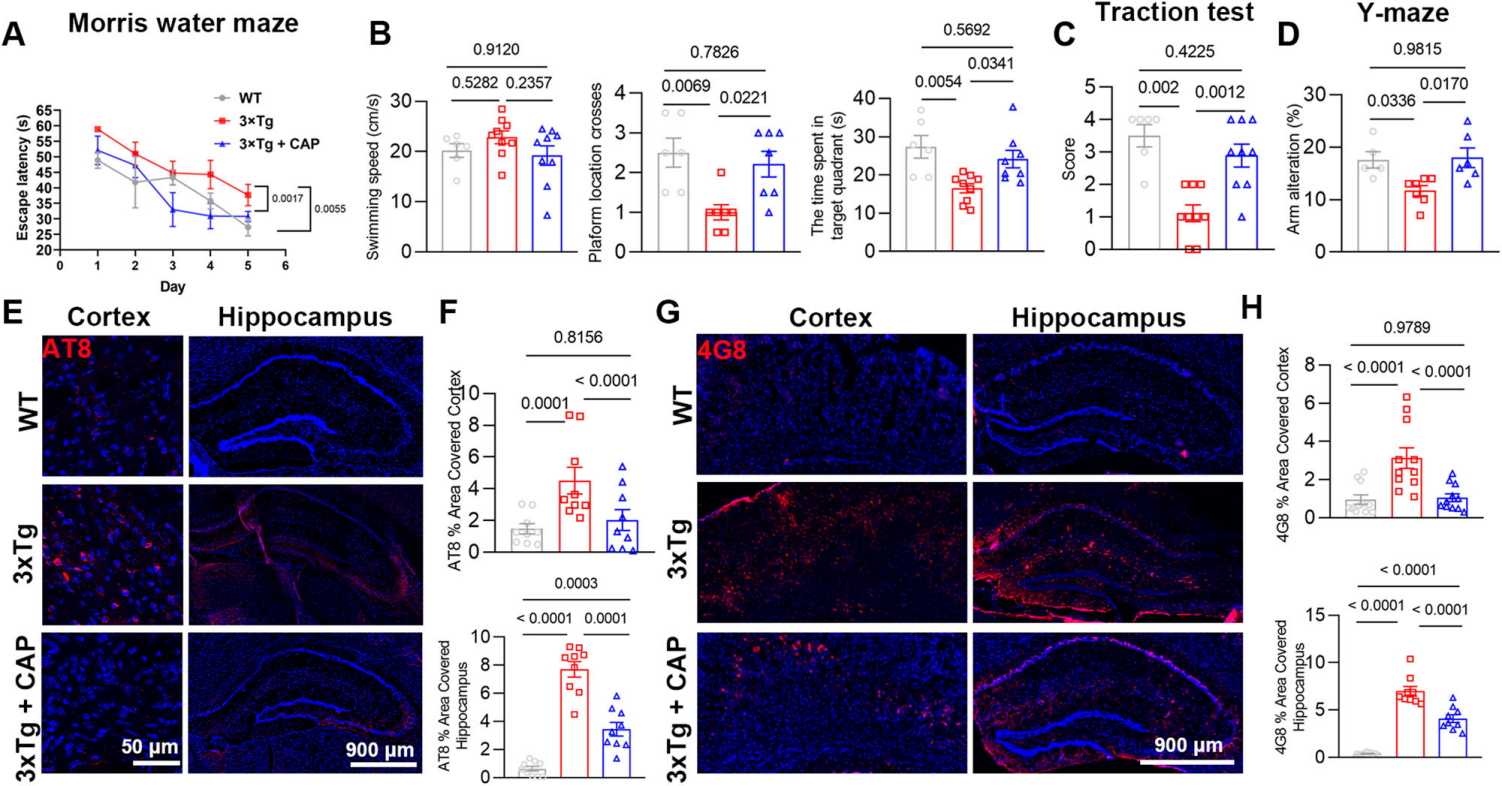
TRPV1 activation rescued memory impairment and attenuated tau pathology in 3xTg mice. **A**, **B** MWM behavioral assessment of WT, 3xTg, 3xTg+CAP mice. **A** Line chart shows MWM escape latency (time to find the hidden platform) for five consecutive days. **B** Histograms show the swimming speed, platform location crosses, and time spent in the target quadrant in the MWM probe trial. **C**, **D** Traction test and Y maze behavioral assessments (*n* = 6, 9, 9 for WT, 3xTg, 3xTg+CAP group, respectively). **E**–**H** Phosphorylated tau (AT-8)-covered area and 4G8 in the cortex and hippocampus of WT, 3xTg, 3xTg+CAP mice (*n* = 5 mice per group). Statistical tests: one-way ANOVA (**A**–**D**, **F**, **H**) followed by Tukey’s post hoc test. Data represent the mean ± s.e.m.

In the MWM, capsaicin treatment significantly improved the learning ability of 3xTg+CAP group mice compared with that of 3xTg mice (Fig. 5A). Capsaicin treatment significantly increased the time in the target quadrant and platform location crosses of 3xTg+CAP group mice compared with 3xTg mice (Fig. 5B). 3xTg mice showed less preference for the traction test than WT mice; this effect was rescued by capsaicin treatment (Fig. 5C). The Y maze test was used to test the spatial working memory functions of mice [42]. Spontaneous alteration analysis showed impaired memory of 3xTg mice compared with WT mice, while 3xTg+CAP group mice spent more time exploring the novel arm than did 3xTg mice (Fig. 5D).

Immunofluorescent images of phosphorylated tau (AT8) (Fig. 5E, F) and 4G8 (Fig. 5G, H) showed significant downregulation in both the cortex and hippocampus of 3xTg+CAP mice compared with 3xTg mice (Fig. 5E–H).

### TRPV1 activation reversed lipid droplet accumulation and inhibited PKM2/SREBP1 axis in the brain of 3xTg mice

**Fig. 6:**
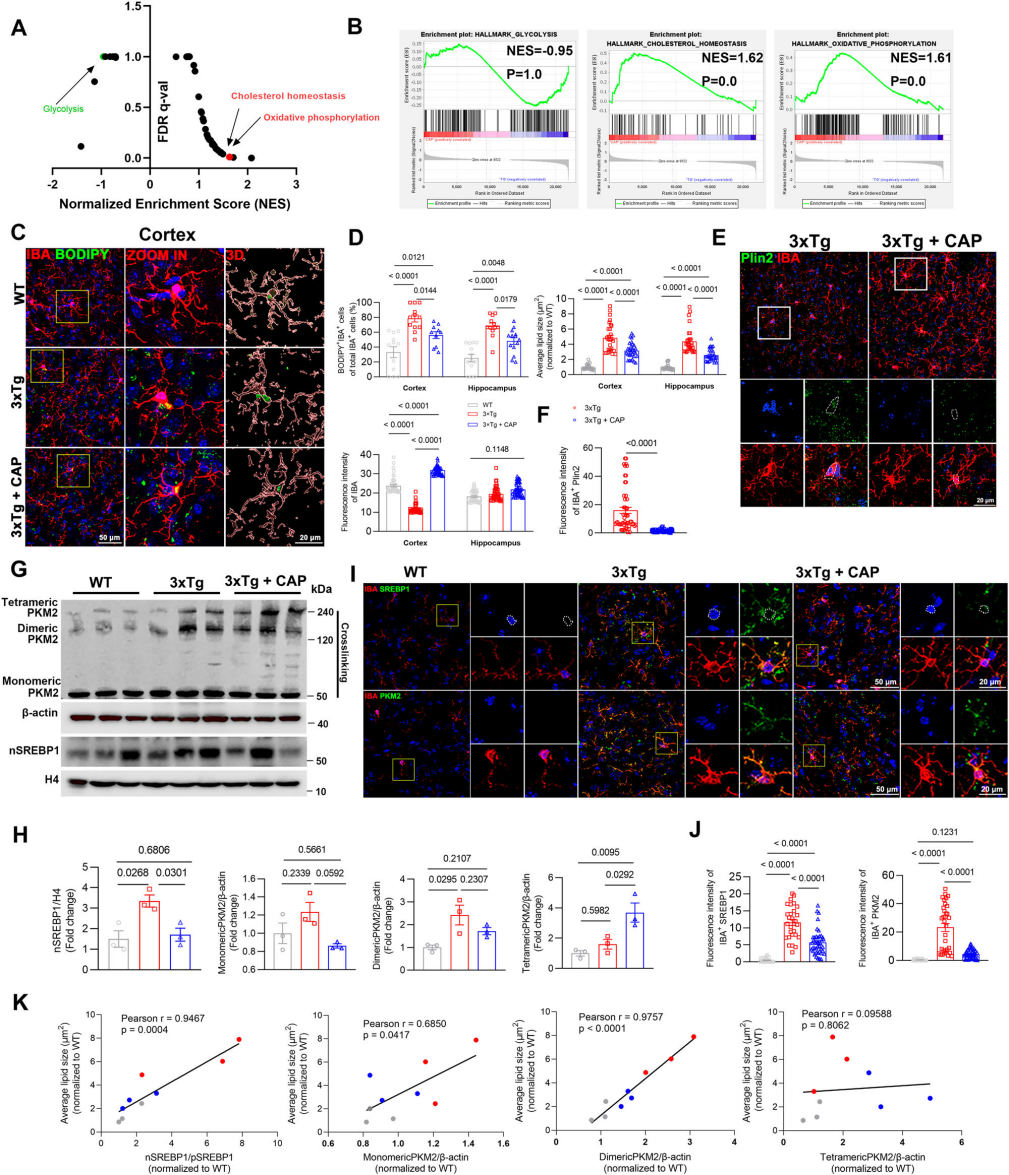
TRPV1 activation reversed lipid droplet accumulation and inhibited PKM2/SREBP1 axis in the brain of 3xTg mice. **A** Gene set enrichment analysis of the top enriched gene signatures associated with 3xTg+CAP versus 3xTg. Hallmark 50 gene sets were used for this analysis. **B** Enrichment plot of glycolysis, cholesterol homeostasis, and oxidative phosphorylation gene in 3xTg+CAP mice brain compared with 3xTg ones by GSEA with adjusted *P*-value and normalized enrichment score (*n* = 3 mice per group). **C**–**F** Representative immunofluorescent images and quantification of BODIPY^+^Iba-1^+^ microglia (C, D) and plin2^+^IBA^+^ microglia (**E**, **F**) in the cerebral cortex of WT, 3xTg, 3xTg+CAP mice (*n* = 5 mice per group). The right panel shows 3D reconstructions of BODIPY^+^IBA^+^ cells. **G**, **H** Western blotting analysis and quantification of nSREBP1, monomeric, dimeric, and tetrameric PKM2 in the cortex of WT, 3xTg and 3xTg+CAP mice (*n* = 3 mice per group). **I**, **J** Representative images and quantification of SREBP1 or PKM2 co-stained with IBA in the cortex of WT, 3xTg, and 3xTg+CAP mice (*n* = 5 mice per group). **K** Correlations between average lipid size and the expression of nSREBP1/pSREBP1, monomeric PKM2, dimeric PKM2, and tetrameric PKM2 in 8 month-old WT, 3xTg, 3xTg+CAP mice. Statistical tests: one-way ANOVA (**D**, **F**, **H**, **J**) followed by Tukey’s post hoc test. Data represent the mean ± s.e.m.

GSEA data showed 3xTg+CAP mice exhibited downregulation of genes associated with glycolysis and upregulated of genes associated with cholesterol homeostasis and oxidative phosphorylation compared with 3xTg mice (Fig. 6A, B). Co-staining of BODIPY or plin2 with Iba-1 showed a significant decrease in lipid droplet accumulation in microglia of 3xTg+CAP mice compared with that in 3xTg mice (Fig. 6C–F).

The expression of nSREBP1, monomeric, dimeric PKM2 was downregulated, while tetrameric PKM2 was upregulated in the cortex of 3xTg+CAP mice (Fig. 6G, H). We co-stained microglia with antibodies against SREBP1 or PKM2 (Fig. 6I, J). 3xTg mouse brain sections showed microglia with strong SREBP1 or PKM2 signals compared to WT sections. Immunofluorescence analysis confirmed that TRPV1 activation with capsaicin reduced the strong co-localization of SREBP1 or PKM2 in microglia of 3xTg+CAP mouse brain sections (Fig. 6I, J). Additionally, through correlation analysis, we found that the average lipid size was positively correlated with the expression of nSREBP1/pSREBP1 and dimeric PKM2, but showed no significant correlation with the expression of tetrameric PKM2 (Fig. 6K).

### TRPV1 activation restored microglia morphology of 3xTg mouse brains

**Fig. 7:**
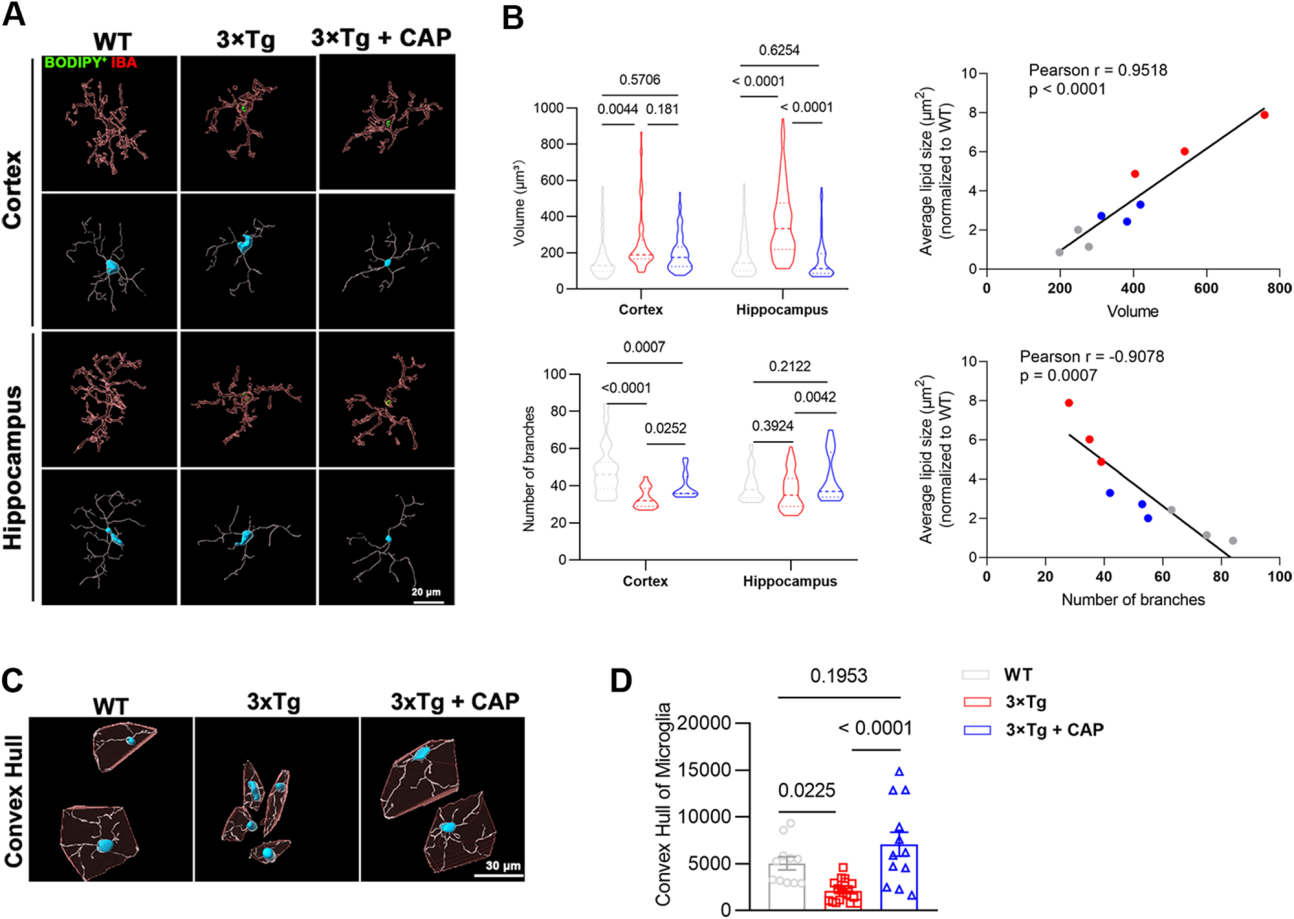
TRPV1 activation attenuated reactive microglia in 3xTg mice. **A** Representative three-dimensional rendering of IBA staining in the cortex and hippocampus of 8 month-old WT, 3xTg, and 3xTg+CAP mice. **B** Quantification of microglial cell volume and the number of branches (*n* = 5 mice per group). Correlations between average lipid size and microglial cell volume, as well as the number of microglial branches, in 8 month-old WT, 3xTg, and 3xTg+CAP mice. **C**, **D** Representative images of 4G8 co-stained with IBA in the cortex and hippocampus of 8-month-old WT, 3xTg, and 3xTg+CAP mice, with quantification of microglia (proximity to 4G8^+^ β-amyloid plaque) morphology (*n* = 5 mice per group). Statistical tests: one-way ANOVA (**B**, **D**) followed by Tukey’s post hoc test. Data represent the mean ± s.e.m.

An Imaris software-based analysis pipeline was used to analyze microglia morphology, allowing the use of highly reproducible machine learning algorithms to quantify differences in single-cell resolution between groups [46]. Three-dimensional reconstructed images of BODIPY^+^ IBA^+^ microglia revealed an increase in cytosolic volume and a decrease in the number of dendritic branches of lipid droplet-accumulated microglia. TRPV1 activation not only reduced the accumulation of microglial lipid droplets in the brains of 3xTg mice, but also restored microglial morphology (Fig. 7A, B). Additionally, through correlation analysis, we found that the average lipid size was positively correlated with the cell volume of microglia and negatively correlated with the number of microglial branches (Fig. 7B). In addition, we analyzed the morphology of plaque-associated microglia in the brains of 3xTg mice. Compared with microglia in the brains of WT mice, plaque-associated microglia of 3xTg mice exhibited smaller convex hull volumes and fewer dendritic branches. Activation of TRPV1 restored morphological features of dysfunctional microglia (Fig. 7C, D).

### TRPV1 activation reversed the transcriptomic changes of inflammation-activated microglia of 3xTg mice

**Fig. 8:**
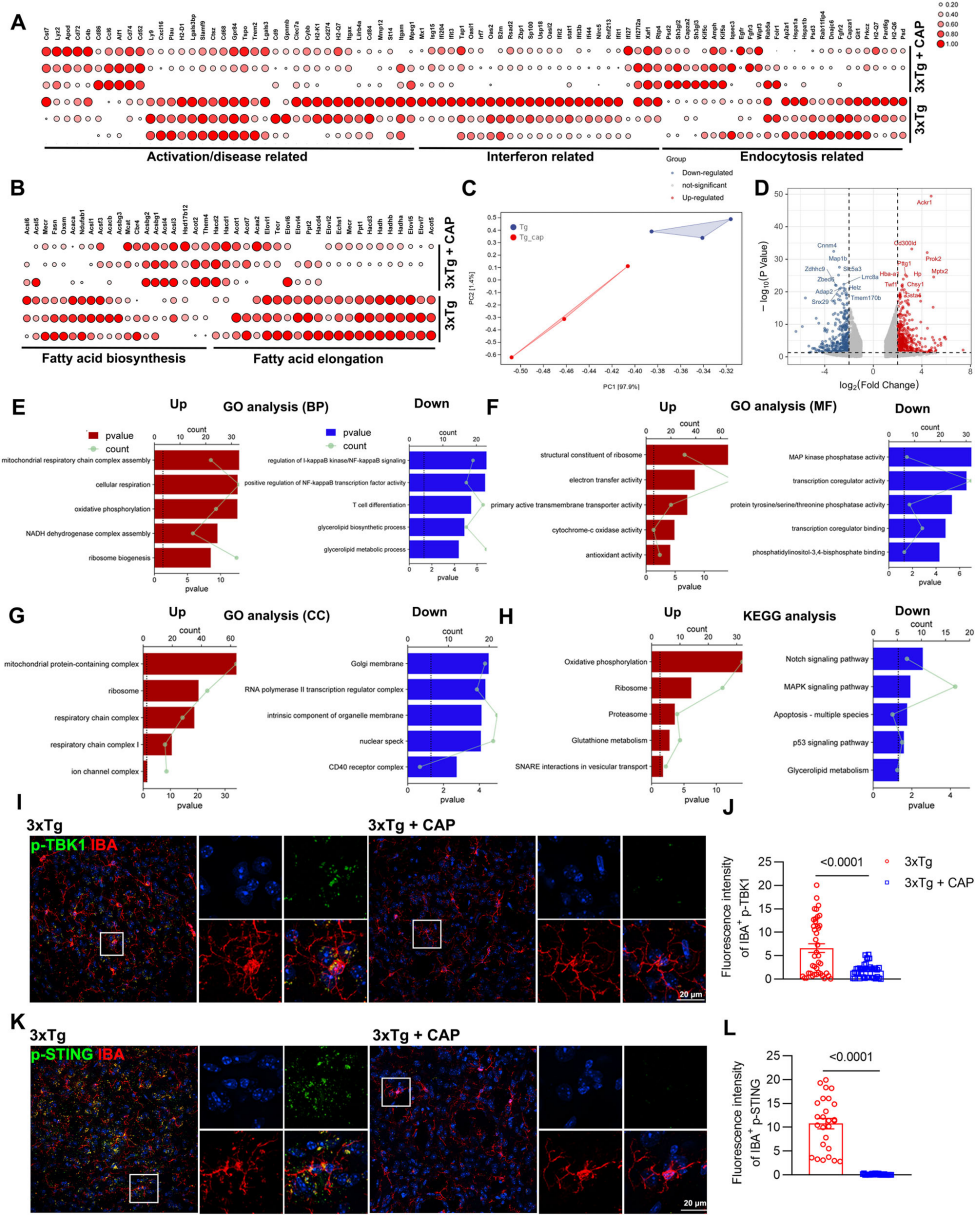
TRPV1 activation reversed the transcriptomic changes of inflammation-activated microglia of 3xTg mice. **A**, **B** Differential gene expression from bulk RNA-seq analysis of microglia isolated from 8 month-old 3xTg and 3xTg+CAP mice (*n* = 3 mice per group). The heatmap shows all significant DEGs associated with interferon or activation/disease-related microglia and lipid metabolism. **C**, **D** Principal component analysis of 8 month-old 3xTg and 3xTg+CAP mice using the whole transcriptome (“all genes”). Volcano plot of the 1561 downregulated DEGs (blue) and 1833 upregulated DEGs (red) of 8 month-old 3xTg and 3xTg+CAP mice (false discovery rate (FDR) ≤ 0.01, | log2[fold change (FC)] | ≥ 2). **E**–**G** The left panel shows the top 5 most enriched GO Biological Process gene sets (**E**) for upregulated DEGs, while the right panel shows those for downregulated DEGs. The same is done for GO Molecular Function gene sets (**F**) and GO Cellular component gene sets (**G**). **H** The left panel shows the top 5 most enriched KEGG gene sets (**H**) for upregulated DEGs, while the right panel shows those for downregulated DEGs. **I**–**L** Representative images and quantification of p-TBK1 (**I**) or p-STING (**K**) co-stained with IBA in the cortex of 8 month-old 3xTg and 3xTg+CAP mice (*n* = 5 mice per group). Statistical tests: two-sided Student’s *t*-test (**J**, **L**). Data represent the mean ± s.e.m.

To uncover potential mechanisms underlying TRPV1 activation in the brain, we performed RNA-seq analysis of microglia isolated from 8-month-old 3xTg and 3xTg+CAP mice. First, the expression of marker genes associated with activation/disease-related or interferon-associated microglia were significantly decreased in microglia of the 3xTg+CAP group compared to the 3xTg group (Fig. 8A). Furthermore, we found that the de novo lipid synthesis and fatty acid elongation pathways were downregulated in microglia of the 3xTg+CAP group while the cellular endocytosis pathway was upregulated (Fig. 8A, B). These results suggest that TRPV1 activation could rescue the dysfunction of inflammation-activated microglia through modulation of lipid metabolism. Transcriptional analysis revealed 3394 significantly DEGs, with 1561 downregulated and 1833 upregulated DEGs (Fig. 8C, D). According to the GO analysis, the upregulated DEGs were enriched in three different GO gene sets related to mitochondrial respiratory chain, oxidative phosphorylation, and ion channel complex (Fig. 8E–G). The downregulated DEGs were enriched in three different GO gene sets related to inflammation activation, immune response, and glycolipid metabolism (Fig. 8E–G). KEGG showed that the upregulated DEGs were enriched in KEGG gene sets related to oxidative phosphorylation and multiple neural synapses (Fig. 8H). The downregulated DEGs were enriched in KEGG gene sets related to the immune system and inflammation response (Fig. 8H). All of this suggests that TRPV1 activation reversed the transcriptomic changes of inflammation-activated microglia of 3xTg mice. To confirm that IFN-associated microglia were significantly decreased in the brains of 3xTg+CAP mice, we performed histological staining with p-TBK1 or p-STING and IBA. The results showed that the intensity of p-TBK1 and p-STING in microglia was downregulated in the brains of 3xTg+CAP mice (Fig. 8I–L).

## Discussion

Here, we report lipid droplet accumulation and neuroinflammation in microglia and neurons in the 3xTg mouse brain. We observed significant upregulation of PKM2 and SREBP1 expression levels, which were predominantly localized in microglia of 3xTg mice. RNA sequencing analysis of microglia isolated from 3xTg mice showed transcriptomic changes in lipid metabolism, innate inflammation, and phagocytosis dysfunction. PKM2 dimerization was necessary for SREBP1 activation and lipogenesis of lipid droplet-accumulating microglia. TRPV1 pharmacological activation with capsaicin attenuated Aβ-induced microglial lipid metabolic dysfunction and restored phagocytic activity by inhibiting PKM2 dimerization and reducing SREBP1 activation in microglia and neurons of 3xTg mice. Capsaicin also rescued neuronal loss, tau pathology, and memory impairment in 3xTg mice. Our study suggests that TRPV1-PKM2-SREBP1 axis regulation of neuronal and microglia lipid metabolism could be a therapeutic approach to alleviate the consequences of the AD.

Cellular abnormal lipid accumulation was recognized as a key characteristic of immune dysfunction in myeloid cells. Lipid droplets, lipid-storing organelles containing neutral lipids such as cholesterol and glycerolipids, are inflammation markers in response to stress and inflammation. Evidence from statins reported in clinical trials have highlighted the relationship between lipid homeostasis dysfunction and AD. Excess brain cholesterol levels are related to amyloid-β deposition, which can alter cholesterol homeostasis. Cholesterol-induced ROS overexpression accelerates the onset of AD neuropathological hallmarks including amyloid-β deposition and tau phosphorylation in the APP/PS1/SREBP2 transgenic mouse model [47].

Dysfunction of fatty acid degradation in astrocytic mitochondria triggers neuroinflammation and neurodegeneration [48]. Lipid-accumulated reactive astrocytes induce neuron oxidative stress, microgliosis via IL-3, and inhibit fatty acid biosynthesis and phospholipids for myelin replenishment. Oxidative phosphorylation, cholesterol homeostasis, and fatty acid metabolism signature were among the top downregulated pathways in 3xTg mice compared with WT mice (Fig. 1A, B).

ApoE4 leads to lipid metabolism dysregulation and intracellular lipid droplet accumulation in microglia from human ApoE-targeted replacement mice, resulting in antigen presentation enhancement and T-cell activation [31]. Lipid droplet accumulation and neuroinflammation were observed in microglia isolated from microglia-specific TRPV1-gene deficient ApoE4 mice [31]. Capsaicin rescued neuronal autophagy, tau pathology, and memory impairment in ApoE4 HFD mice. TRPV1 activation attenuated microglial lipid droplets, phagocytosis of synapses, MHC-II dependent antigen presentation, and T-cell activation in ApoE4 HFD mice. TRPV1 pharmacological activation with capsaicin attenuated Aβ-tolerant microglial metabolic dysfunction via AKT/mechanistic pathway of rapamycin kinase pathway activity and restored phagocytic activity and autophagy function [27]. TRPV1 activation alleviates cognitive and synaptic plasticity impairments by inhibiting AMPAR endocytosis in the APP23/PS45 mouse model of AD [49].

PKM2 links aerobic glycolysis and inflammatory dysfunction in atherosclerotic coronary artery disease. Oxidized low-density lipoprotein treatment induced phosphorylation of PKM2, promoting nuclear localization by inhibiting PKM2 tetramer formation [16]. The monomer or dimer form of PKM2 translocates to the nucleus and induces expression of secretory IL-1β and pro-glycolytic enzymes such as lactate dehydrogenase, lactate, and glucose transporter member 1. The positive feedback loop of glycolysis/H4K12lα/PKM2 exacerbates dysfunction of microglia in AD [28]. Microglia-specific deletion or pharmacologic inhibition of PKM2 with attenuated reactive microglia improved spatial learning and memory in 5xFAD AD mice. PKM2 dimerization was necessary for SREBP1 activation and lipogenesis in BV2 cells (Fig. 4A–F). Capsaicin promotes cholesterol efflux via upregulation of the expression of ABCA1 and ABCG1 in macrophages of atherosclerosis [50]. Capsaicin impeded oxidized low-density lipoprotein-induced foam cell formation via autophagy in vascular smooth muscle cells. Capsaicin also blocked the expression of dimeric PKM2 and nSREBP1 in the presence of Aβ_1-42_ (Fig. 4A, B).

PKM2 is upregulated in activated immune cells, platelets, and smooth muscle cells, which has generated significant interest [10–13]. PKM2 possesses protein kinase activity in addition to its role in glycolysis. Dimeric PKM2 translocates to the nucleus to catalyze the transfer of phosphate of PEP to tyrosine, threonine, or serine residues on target substrates [15–18]. Dimeric PKM2 is reported to promote inflammatory activation of macrophages [12–19], allergic airways disease [20], and autoimmune encephalomyelitis [7].

Together, we showed SREBP1 activation-mediated lipid droplet accumulation and neuroinflammation in microglia in 3 x Tg mice brain. TRPV1 pharmacological activation with capsaicin attenuated Aβ-induced microglial lipid metabolic dysfunction via inhibition of PKM2-SREBP1 pathway activity and restored microglial phagocytic activity. In the future, targeting the TRPV1-PKM2-SREBP1 axis might represent an attractive approach to decrease immune inflammation and to restore brain homeostasis in neurodegeneration.

Our study also has several limitations that warrant further investigation. Although we used both 8 month-old male and female 3 x Tg mice in an ~1:1 ratio and did not find a sex-dependent influence of PKM2-SREBP1 in regulating microglial lipid droplet accumulation and function, it is still essential to explore potential gender-specific effects in older mice. Additionally, examining the dose-response curve of capsaicin and conducting longitudinal studies on the duration of capsaicin treatment would be valuable for optimizing the balance between efficacy and safety in potential clinical applications.

## Supplementary information

Full and uncropped western blots

Supplementary figure and table legends

Table S1

## Data Availability

The datasets used and analyzed during the current study are available from the corresponding author on reasonable request.
