# Supplementary figures and images for: The TRPV1-PKM2-SREBP1 axis maintains microglial lipid homeostasis in Alzheimer’s disease
Source: Cell Death Dis. 2025 Jan 14;16(1):14. doi: 10.1038/s41419-024-07328-8 (PMC11732990; doi:10.1038/s41419-024-07328-8)

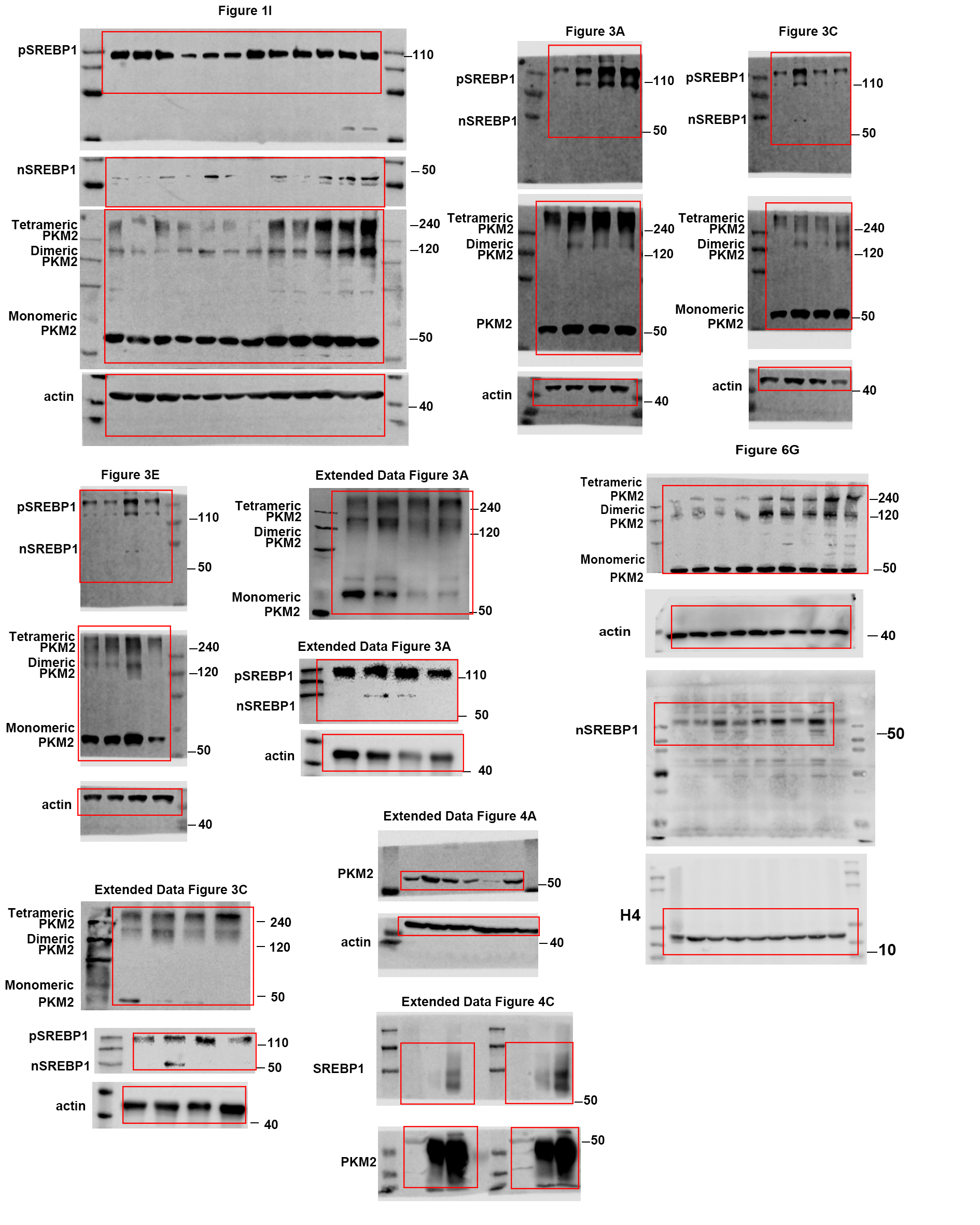

Supplement: Supplementary file 1 — Full and uncropped western blots [file 41419_2024_7328_MOESM1_ESM.tif]
