# Supplementary material for: The TRPV1-PKM2-SREBP1 axis maintains microglial lipid homeostasis in Alzheimer’s disease
Source: Cell Death Dis. 2025 Jan 14;16(1):14. doi: 10.1038/s41419-024-07328-8 (PMC11732990; doi:10.1038/s41419-024-07328-8)
Supplement: Supplementary file 2 — Supplementary figure and table legends [file 41419_2024_7328_MOESM2_ESM.docx]

**Extended Data Figure legend**


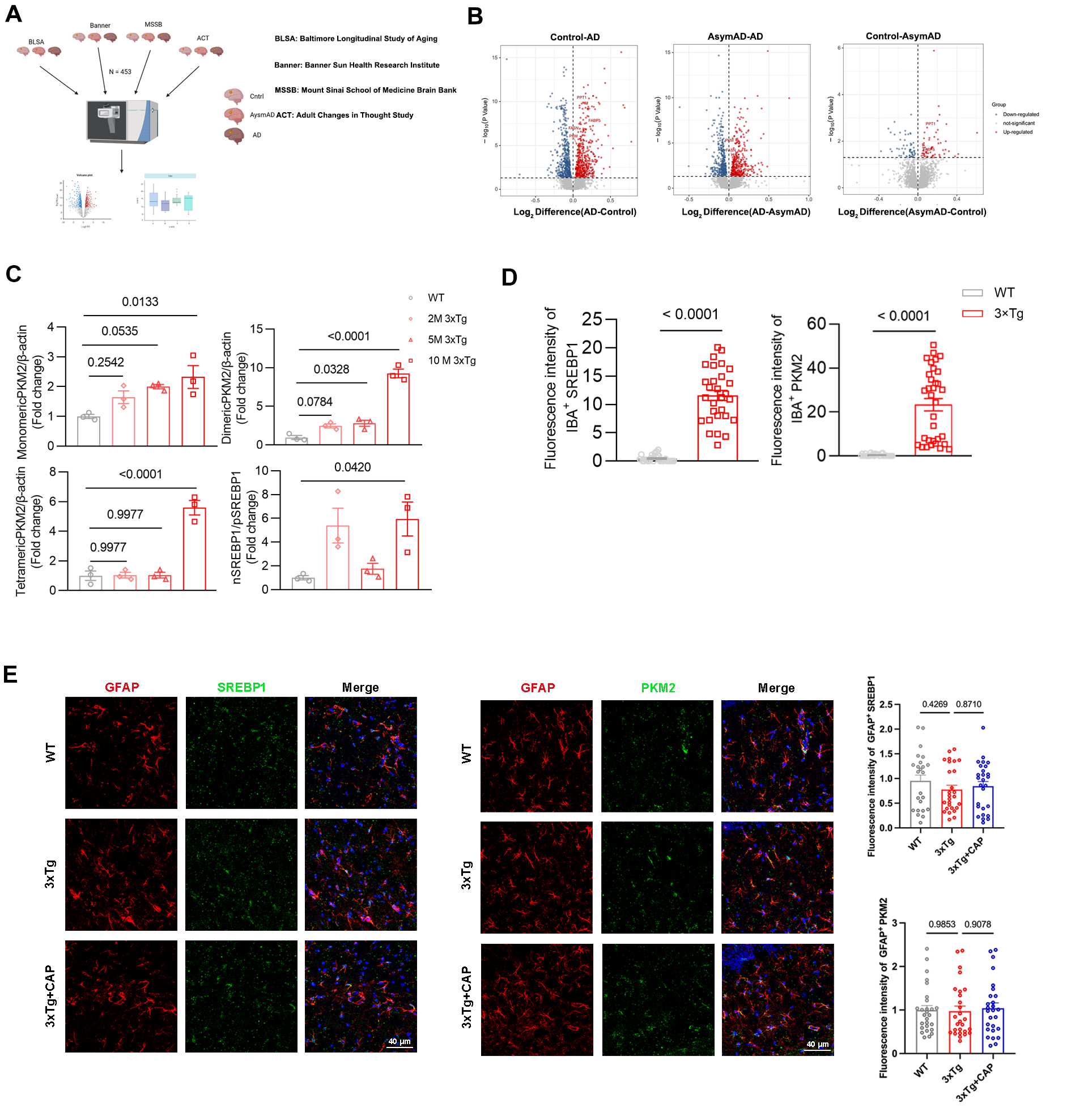


**Extended Data Figure 1. Correlation of lipid droplet accumulation and expression of PKM2/SREBP1 axis in AD microglia.**

A Schematic diagram of the public Large-scale proteomic analysis. B Differences in protein abundance of AD versus control, AD versus AsymAD, and AsymAD versus control brains are expressed as fold change versus t-statistics for the given comparison (AD = 230, AsymAD = 98, AsymAD = 91). C Quantification of protein levels (n = 3 mice per group). D Quantification of pkm2 and SREBP1 intensity in microglia (n > 30 cells per group). E Representative images of SREBP1 and PKM2 co-stained with GFAP in the cortex of mice, along with quantification of PKM2 and SREBP1 intensity in astrocytes (n = 3 mice per group). AsymAD: asymptomatic AD; ACT: Adult Changes in Thought Study; Banner: Banner Sun Health Research Institute; BLSA: Baltimore Longitudinal Study of Aging; MSSB: Mount Sinai School of Medicine Brain Bank. Statistical tests: one-way ANOVA (C-E) followed by Tukey’s post hoc test. Data represent the mean ± s.e.m.


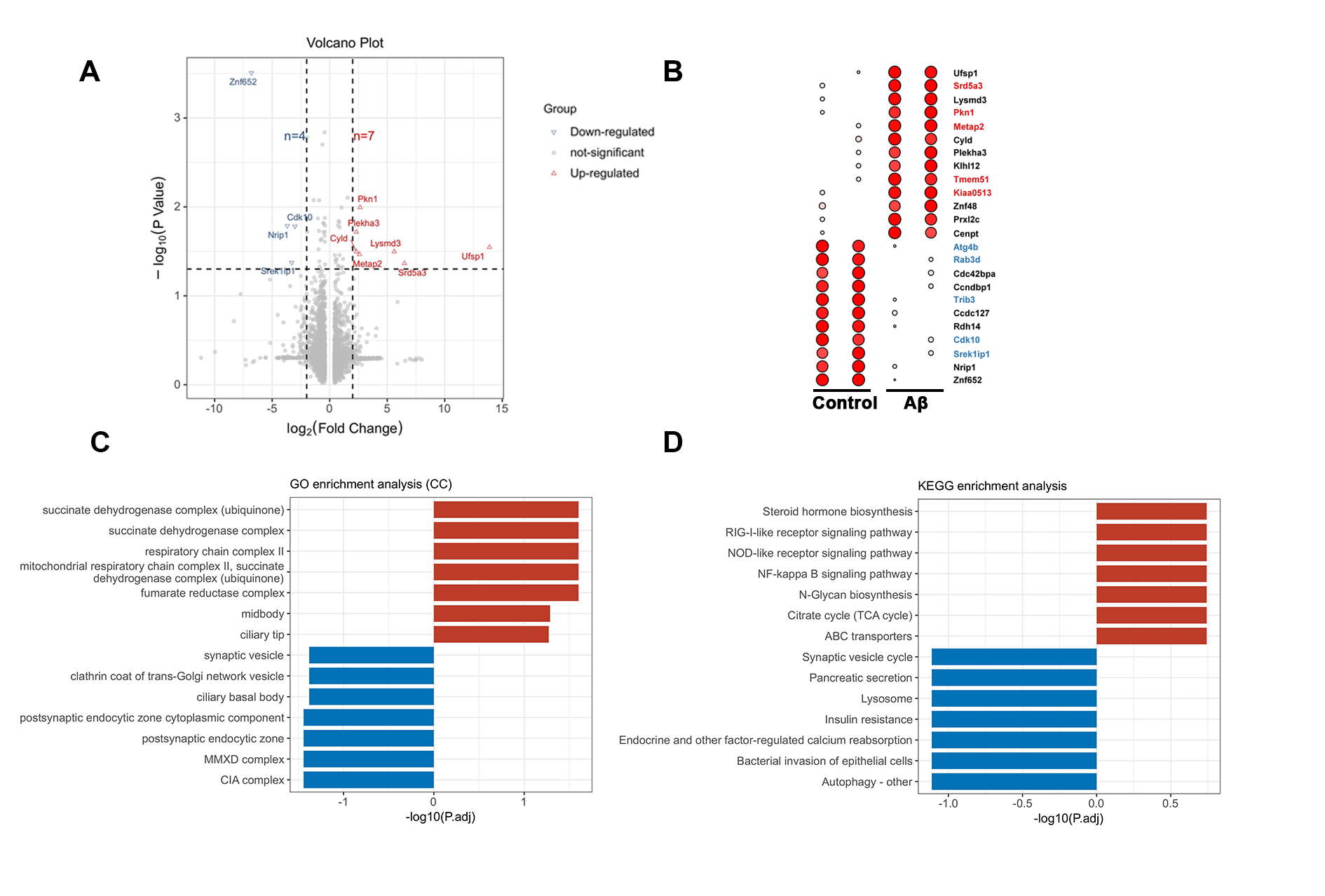


**Extended Data Figure 2. Protein analysis of Aβ-induced BV2 cells.**

A, B Volcano plot of Control and Aβ-induced BV2 cells (false discovery rate (FDR) ≤ 0.01, | log_2_[fold change (FC)] | ≥ 2). The heatmap shows all significant DEPs associated with inflammation and lipid metabolism. C The upper red panel shows the top 7 most enriched GO Cellular Component gene sets for upregulated DEPs, while the lower blue panel for downregulated DEPs. D The left panel shows the top 7 most enriched KEGG gene sets for upregulated DEPs, while the right panel shows those for downregulated DEPs.

**
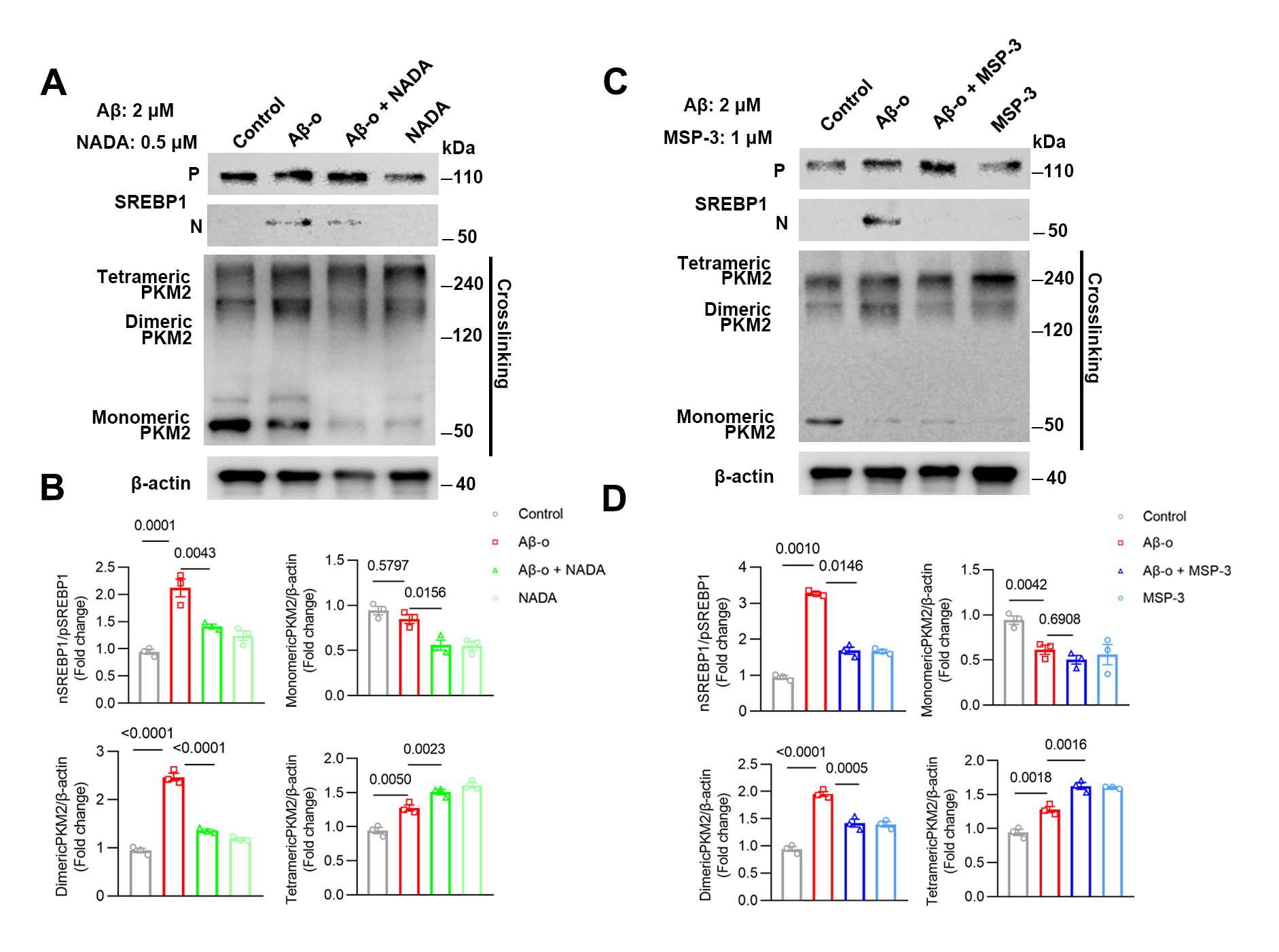
**

**Extended Data Figure 3. TRPV1/PKM2/SREBP1 axis in Aβ_1-42_-stimulated BV2 cells.**

A-D Western blotting analysis and quantification of nSREBP1/pSREBP1, monomeric, dimeric, and tetrameric PKM2 in 2 μM Aβ_1-42-_stimulated BV2 cells for 24 h with 0.5 μM NADA (A, B) or 1 μM MSP-3 (C, D) pretreatment. Statistical tests: one-way ANOVA (B, D) followed by Tukey’s post hoc test. Data represent the mean ± s.e.m.


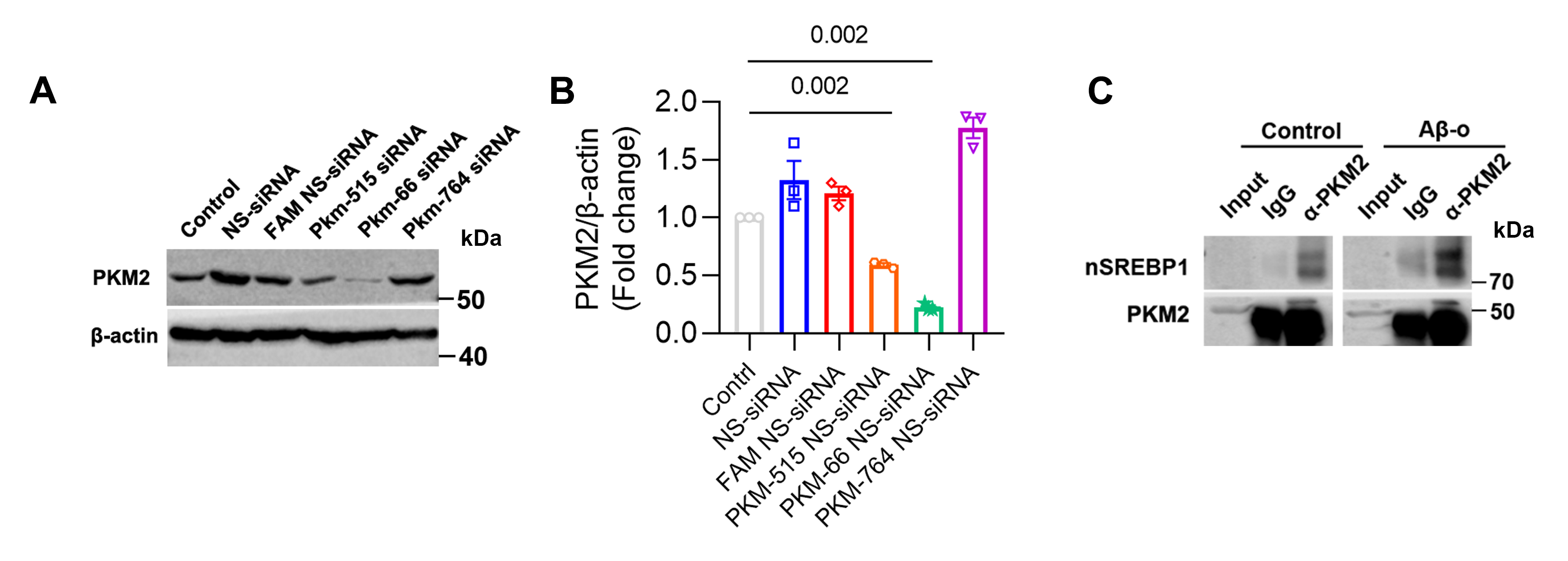


**Extended Data Figure 4.** **TRPV1/PKM2/SREBP1 axis in Aβ_1-42_-stimulated BV2 cells.**

A, B Western blotting analysis of the effects of PKM2 knockdown in BV2 cells by three independent siRNA (NS-siRNA as control), with quantification (B) of protein levels. C Immunoprecipitation analysis of endogenous PKM2 binding to an endogenous nuclear form of SREBP1 in BV2 cells stimulated without or with Aβ. Statistical tests: one-way ANOVA (B) followed by Tukey’s post hoc test. Data represent the mean ± s.e.m.


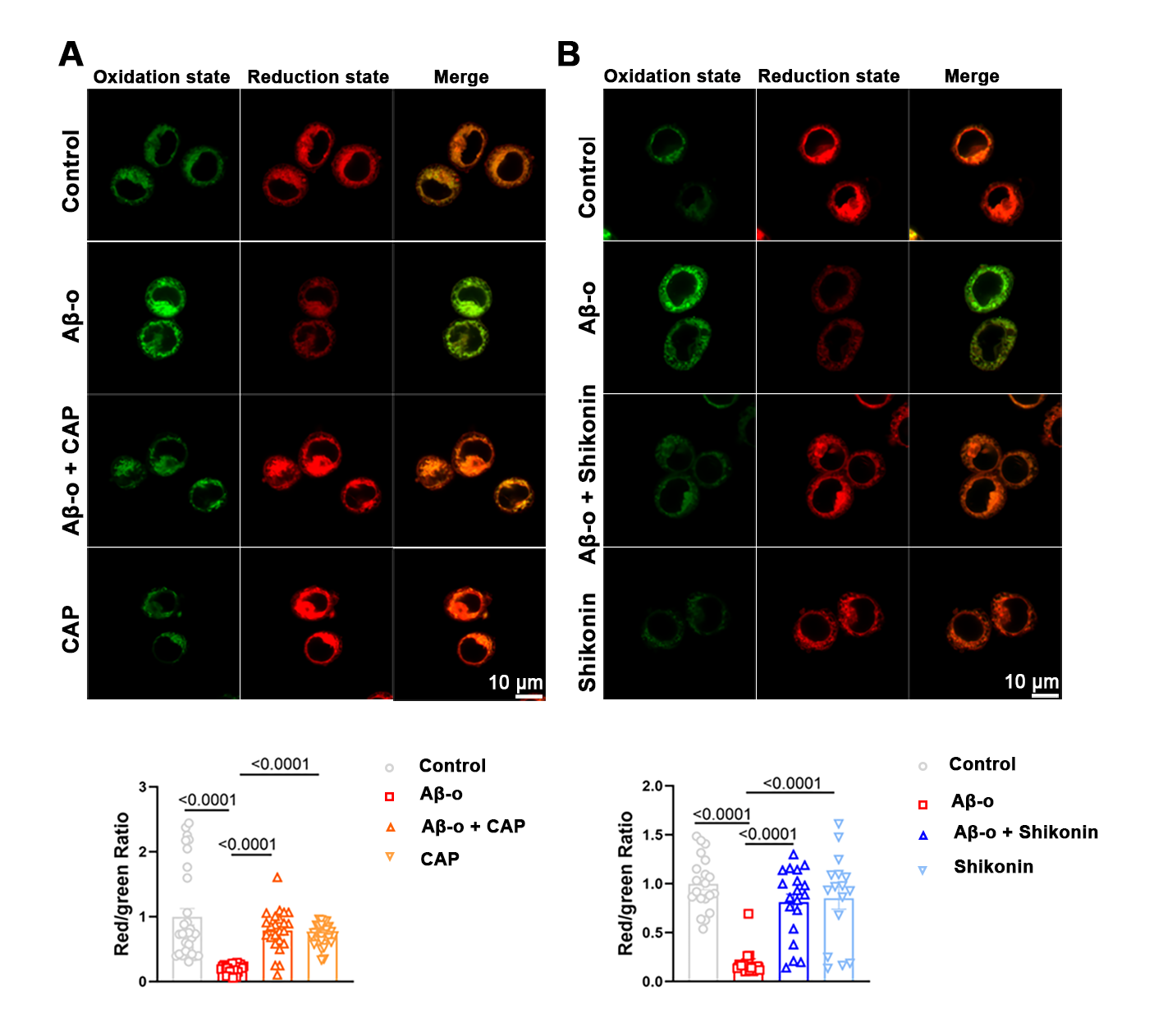


**Extended Data Figure 5. TRPV1/PKM2/SREBP1 axis regulates lipid peroxidation in Aβ_1-42_-stimulated BV2 cells**

Representative images of 2 μM Aβ_1-42_-stimulated BV2 cells for 24 h with 10 μM capsaicin (A) or 1 μM shikonin (B) pretreatment. A, B Lipid peroxidation was measured by C11-BODIPY staining (n = 3, biological replicates). Statistical tests: one-way ANOVA followed by Tukey’s post hoc test. Data represent the mean ± s.e.m.
