# Supplementary material for: The TRPV1-PKM2-SREBP1 axis maintains microglial lipid homeostasis in Alzheimer’s disease
Source: Cell Death Dis. 2025 Jan 14;16(1):14. doi: 10.1038/s41419-024-07328-8 (PMC11732990; doi:10.1038/s41419-024-07328-8)
Supplement: Supplementary file 3 — Table S1 [file 41419_2024_7328_MOESM3_ESM.docx]

Table S1. Oligonucleotides and siRNA used in this study

| **Oligonucleotides** | **SOURCE** | **IDENTIFIER** |
| --- | --- | --- |
| **mouse *Acaca* FWD: AATGAACGTGCAATCCGATTTG**  **mouse *Acaca* REV: ACTCCACATTTGCGTAATTGTTG** | **Sango Biotech** | **N/A** |
| **mouse *Acly* FWD: ACCCTTTCACTGGGGATCACA**  **mouse *Acly* REV: GACAGGGATCAGGATTTCCTTG** | **Sango Biotech** | **N/A** |
| **mouse *Elovl6* FWD: GAAAAGCAGTTCAACGAGAACG**  **mouse *Elovl6* REV: AGATGCCGACCACCAAAGATA** | **Sango Biotech** | **N/A** |
| **mouse *Fasn* FWD: GGAGGTGGTGATAGCCGGTAT**  **mouse *Fasn* REV: TGGGTAATCCATAGAGCCCAG** | **Sango Biotech** | **N/A** |
| **mouse *Hmgcr* FWD: AGCTTGCCCGAATTGTATGTG**  **mouse *Hmgcr* REV: TCTGTTGTGAACCATGTGACTTC** | **Sango Biotech** | **N/A** |
| **mouse *pkm2* FWD: GCCGCCTGGACATTGACTC**  **mouse *pkm2* REV: CCATGAGAGAAATTCAGCCGAG** | **Sango Biotech** | **N/A** |
| **siRNA** |  |  |
| **Pkm-66-Mouse FWD: GGCUGACACCUUCCUGGAACATT**  **Pkm-66-Mouse REV: UGUUCCAGGAAGGUGUCAGCCTT** | **Obio technology** | **N/A** |
| **Pkm-515-Mouse FWD: GCAAGAUCUACGUGGACGAUGTT**  **Pkm-515-Mouse REV: CAUCGUCCACGUAGAUCUUGCTT** | **Obio technology** | **N/A** |
| **Pkm-764-Mouse FWD: GGAAGGUGCUGGGAGAGAAGGTT**  **Pkm-764-Mouse REV: CCUUCUCUCCCAGCACCUUCCTT** | **Obio technology** | **N/A** |
| **Negative control FWD: UUCUCCGAACGUGUCACGUTT**  **Negative control REV: ACGUGACACGUUCGGAGAATT** | **Obio technology** | **N/A** |
| **Negative control FAM FWD: UUCUCCGAACGUGUCACGUTT**  **Negative control FAM REV: ACGUGACACGUUCGGAGAATT** | **Obio technology** | **N/A** |
